# Long-term health outcomes and comorbidities in cytochrome P450 oxidoreductase (POR) deficiency: a scoping review of evidence and knowledge gaps

**DOI:** 10.1530/EC-26-0354

**Published:** 2026-10-15

**Authors:** Chrysanthi Kouri, Asmitha Thirukkumar, Christa E Flück

**Affiliations:** ^1^ Pediatric Endocrinology, Diabetology and Metabolism, Department of Pediatrics, Inselspital, Bern University Hospital, University of Bern, Bern, Switzerland; ^2^ Department for BioMedical Research, University of Bern, Bern, Switzerland; ^3^ Faculty of Medicine, University of Bern, Bern, Switzerland

**Keywords:** P450 oxidoreductase deficiency, cytochrome P450 enzymes, long-term outcomes, comorbidities, steroidogenesis disorders

## Abstract

**Graphical Abstract:**

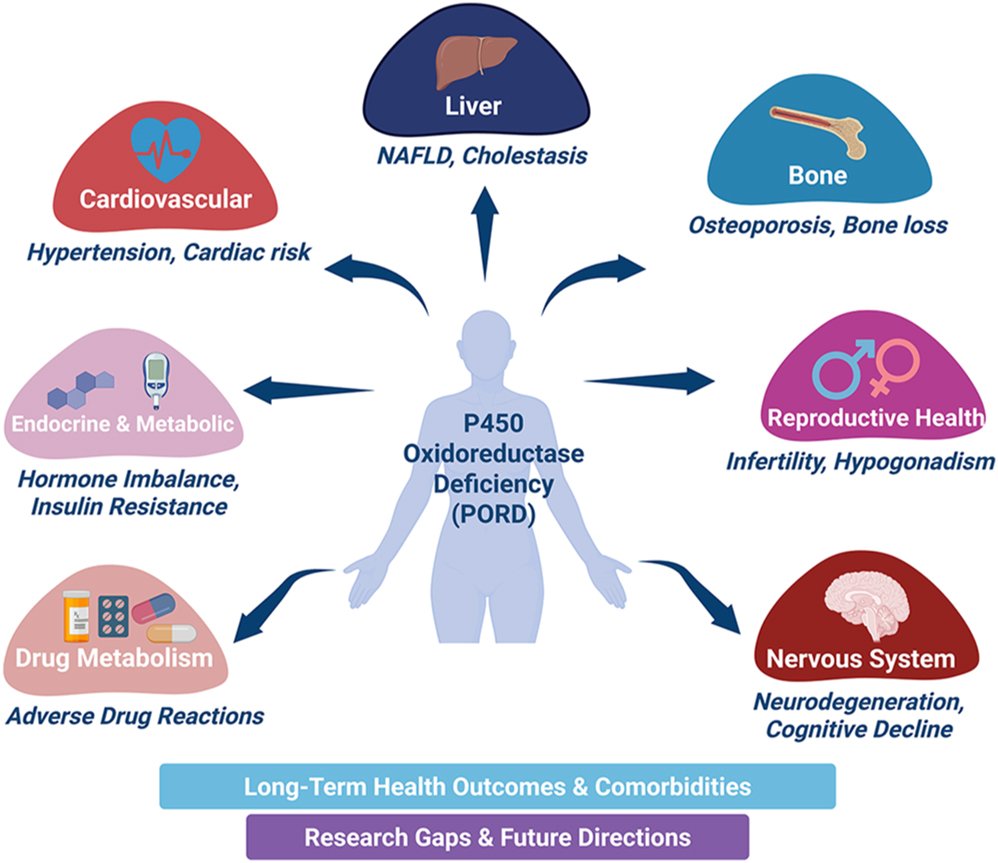

**Abstract:**

P450 oxidoreductase deficiency (PORD) is a rare congenital disorder of steroidogenesis caused by pathogenic variants in POR, the obligate electron donor to all microsomal cytochrome P450 (CYP) enzymes. While the early clinical presentation of PORD – characterized by adrenal insufficiency, differences of sex development, and Antley–Bixler-like skeletal malformations – is well established, its long-term consequences remain poorly defined. This scoping review synthesizes current evidence on multisystem outcomes in PORD and integrates insights from the broader biology of POR-dependent enzymes to inform potential long-term risks. Across 51 known POR-partner enzymes, numerous polymorphisms have been associated with cardiovascular, metabolic, reproductive, skeletal, neurological, inflammatory, and oncological phenotypes in the general population. Although many of these conditions have not yet been reported in PORD, their mechanistic plausibility highlights important gaps in surveillance. Emerging clinical observations, including hypertension, altered drug metabolism, impaired fertility, and reduced bone health, further underscore the need for systematic long-term follow-up. Experimental models additionally suggest vulnerabilities related to lipid accumulation, bile acid dysregulation, and hepatocellular injury. Despite these concerns, no evidence-based guidelines exist for the lifelong management of PORD, and psychosocial outcomes remain entirely unexplored. Collectively, the available data indicate that PORD is a multisystem disorder with potential age-dependent manifestations extending beyond childhood. Prospective longitudinal studies, mechanistic investigations, and coordinated international registry efforts are essential to define the true long-term phenotype and to develop standardized monitoring and management strategies for affected individuals.

**Lay summary:**

P450 oxidoreductase deficiency (PORD) is a rare genetic condition that affects many body systems because the POR enzyme supports dozens of important metabolic pathways. Most research has focused on early symptoms, such as hormone problems, differences in sex development, and bone abnormalities. Much less is known about how PORD affects people later in life. This review brings together current evidence and highlights possible long-term risks, including high blood pressure, liver and metabolic problems, reduced fertility, and bone health issues. Longer-term studies are urgently needed to understand these risks and to guide better care for individuals with PORD.

## Introduction

Cytochrome P450 oxidoreductase (POR) is a microsomal flavoprotein located in the endoplasmic reticulum and serves as the obligatory electron donor to all microsomal cytochrome P450 enzymes (CYPs) and several non-P450 enzymes involved in steroidogenesis, cholesterol synthesis, and xenobiotic metabolism (1). Biallelic pathogenic variants in POR cause cytochrome P450 oxidoreductase deficiency (PORD) (2, 3), a rare form of congenital adrenal hyperplasia (CAH) first described by Peterson as mixed oxidase disease (4). Impaired electron transfer to the steroidogenic enzymes CYP17A1, CYP21A2, and CYP19A1 disrupts glucocorticoid, mineralocorticoid, and sex steroid biosynthesis (5).

PORD is characterized by a broad and heterogeneous clinical spectrum (5, 6). The more severe, classic, early-onset form typically presents in infancy with differences of sex development (DSD), primary adrenal insufficiency (PAI), and skeletal malformations resembling Antley–Bixler syndrome (ABS) (7). Milder forms may first become evident later, e.g. during puberty, often with delayed or absent pubertal development (8, 9). A non-classic, late-onset form has been described in adult women with primary or secondary amenorrhea or menstrual disturbances resembling polycystic ovary syndrome (PCOS) (2, 6, 9). This phenotypic variability reflects the diverse interactions between POR and its multiple CYP partners, each of which may be differentially affected by specific POR variants.

The multisystem involvement of PORD is increasingly recognized. In PORD, cortisol deficiency results predominantly from impaired 21-hydroxylase activity (CYP21A2) and, to a lesser extent, from impaired 17-hydroxylase activity (CYP17A1). Primary adrenal insufficiency (PAI) is present in approximately three-quarters of patients (10). Although usually partial, it can still precipitate adrenal crises during severe physiological stress, such as infections or sepsis. DSDs are also frequent, affecting roughly 70% of individuals (10, 11). In 46,XY patients, DSD primarily results from diminished CYP17A1 activity and consequent impairment of fetal testicular androgen production. In 46,XX fetuses, DSD and maternal virilization during pregnancy arise from impaired CYP21A2, CYP17A1, and CYP19A1 activities. Accumulation of androgenic precursors and their metabolism via both the classic and alternative (‘backdoor’) pathways contribute to androgen excess, while impaired aromatase (CYP19A1) activity in the fetal–placental unit prevents conversion of fetal androgens to estrogens (8, 12, 13). Large ovarian cysts, often prone to spontaneous rupture, are a common finding in females with PORD (39%; (10)). They are thought to result from elevated gonadotropins combined with impaired CYP51A1 activity, leading to oocyte arrest (9). Consequently, delayed or absent puberty and infertility have been reported in several PORD patients because of disturbed steroidogenesis (6, 9). Skeletal malformations are very common in PORD (84%; (10, 11). These abnormalities arise from impaired activity of enzymes involved in cholesterol synthesis within chondrocytes, including 14α-lanosterol demethylase (CYP51A1) and squalene epoxidase (SQLE, a non-P450 enzyme) (5, 14). In addition, enzymes involved in retinoic acid metabolism, such as CYP26B1 and CYP26A1, depend on POR for electron transfer, and their impaired activity contributes to skeletal defects (15, 16).

Although more than 100 patients with PORD have been reported, the extent of multisystem involvement and long-term health implications remains insufficiently characterized. Existing literature primarily focuses on adrenal, reproductive, and skeletal manifestations, while the broader consequences of POR dysfunction, given its pleiotropic role across numerous enzymatic pathways, are not well defined. Furthermore, the functional relevance of many POR-dependent enzymes in the context of PORD is unclear. Studies demonstrate that POR variants can impair *in vitro* activities of drug-metabolizing CYPs and alter *in vivo* drug clearance in a variant- and substrate-specific manner (17, 18, 19, 20, 21, 22, 23). POR variants may also mimic deficiencies of individual CYP enzymes (24). Investigating gene–disease associations of POR partners may provide insight into possible, so far hidden, phenotypes that could also emerge from PORD and thereby help in understanding the phenotypic variability in PORD. In addition, polymorphisms in POR partners might modulate PORD variability or cause comorbidities that have not yet been explained. Given these uncertainties, a comprehensive mapping of the existing clinical evidence and the biologically suspected broader impact is needed. A scoping review is particularly suited to i) systematically identify and collate all reported cases of PORD, ii) characterize the range of clinical manifestations and comorbidities, iii) summarize long-term outcomes, and iv) explore phenotypes associated with genetic variation in POR-partner enzymes that may also inform the broader phenotypic spectrum of PORD.

This scoping review therefore aims to synthesize all published reports of PORD from 2004 onward, with a focus on comorbidities and long-term outcomes, and to contextualize these findings by examining reported phenotypes associated with genetic variation in POR-partner genes. This approach will help clarify the potential multisystem impact of POR dysfunction and identify gaps to guide future research and clinical care.

## Methods

A comprehensive literature search was conducted on September 16, 2025, in Ovid MEDLINE, Embase, and PubMed to identify publications reporting patients with cytochrome P450 oxidoreductase deficiency (PORD). All databases were searched from 2004 (e.g. the first genetic description of PORD (2)) to September 2025. The Preferred Reporting Items for Systematic Reviews and Meta-Analyses (PRISMA) guidelines for scoping reviews were followed to conduct this literature search (25). The detailed selection process is presented in Supplementary Fig. S1 (see section on Supplementary materials given at the end of the article) according to PRISMA guidelines.

In Ovid MEDLINE, the search strategy combined the terms ‘congenital adrenal hyperplasia, ambiguous genitalia and differences/disorders of sex development’ with ‘cytochrome P450 oxidoreductase’, ‘POR gene’, ‘POR deficiency’, or ‘cytochrome P450 oxidoreductase deficiency’, yielding 254 articles. In Embase, the term ‘P450 oxidoreductase deficiency’ was used together with the filters ‘disease: PORD’ and ‘review’, identifying 43 articles. In PubMed, the same core term was applied with filters for review, systematic review, humans, English, full text, and a publication date range of 2004–2025, resulting in 136 articles.

Articles were included if they met the following criteria: i) the patients had genetically confirmed biallelic P450 oxidoreductase deficiency; ii) the research discussed clinical outcomes, associated morbidities, or long-term health outcomes linked to PORD; iii) the articles were original research articles, clinical or observational studies, case reports, or narrative or systematic reviews; and iv) they were published between 2004 and September 2025 in English or with an English translation available.

Articles were excluded if they met any of the following criteria: i) they focused primarily on other disorders of sex development without specific mention or focus on PORD; ii) they were based solely on animal models or *in vitro* research without direct clinical relevance to human PORD; or iii) clinically relevant data could not be reliably attributed to individual patients.

Altogether, 433 records were retrieved. After removal of duplicates, 303 unique articles remained for screening. One author screened the titles and abstracts and then assessed the full texts of 113 potentially eligible articles against the predefined inclusion criteria together with a second author. In total, 54 articles met the inclusion criteria and were included in the review. Articles were excluded primarily because they did not exclusively report on PORD or lacked sufficient clinical information (Supplementary Fig. S1).

Data from all eligible publications were extracted and tabulated by one author and reviewed by the two others (Supplementary Table S1). Extracted variables included current age or age at referral, karyotype, POR genotype, adrenal function, presence of DSD at birth, pubertal development, reproductive health, skeletal findings including bone scores and Antley–Bixler-like features, management, and any additional comorbidities or relevant clinical observations.

To assess the frequency and the impact of specific POR variants on different organ systems, we extracted the specific information from the selected reports and Supplementary Table S1 into a phenotype matrix and constructed a heatmap.

To explore potential phenotypes associated with POR-dependent pathways, LitVar2 and the Online Mendelian inheritance in man (OMIM) database were queried for all 50 known POR-partner genes (Supplementary Table S2). Publications reporting polymorphisms or pathogenic variants associated with disease susceptibility or clinical phenotypes were identified and reviewed. These findings were used to contextualize the possible broader phenotypic spectrum that may arise from POR dysfunction and to support interpretation of the multisystem involvement observed in PORD.

## Results

### Characteristic clinical profile of reported individuals with PORD

A total of 136 patients with PORD were identified from 54 eligible articles included in this scoping review. Among these, 103 (76%) presented with DSD, 103 (76%) with skeletal malformations, and 87 (64%) with PAI (Supplementary Table S1). Forty-nine patients (36%) exhibited all three features concurrently. Three patients had isolated DSD, seven had isolated PAI, and five had isolated skeletal malformations. Thirty-four patients had both DSD and skeletal malformations, 16 had DSD and AI, and 15 had PAI and skeletal malformations. Seven patients did not present with any of these three manifestations; however, six of them had menstrual irregularities and ovarian cysts, and the remaining case was a fetus diagnosed prenatally because of a positive family history and aborted at 24 weeks.

Among the 103 patients with DSD, 38% (39/103) had a 46,XY karyotype and 62% (64/103) had a 46,XX karyotype. The phenotype predominantly included ambiguous genitalia, such as clitoromegaly, partial labial fusion, urogenital sinus, hypospadias, cryptorchidism, micropenis, bifid scrotum, or imperforate anus.

Skeletal malformations in these 103 patients were mainly features of the ABS spectrum. Thirty-eight patients (37%) had a mild ABS phenotype, 47 (46%) had a moderate phenotype, and 18 (17%) had a severe phenotype. ABS severity was defined according to the PORD malformation score described by Krone *et al.* (26). Frequently reported skeletal malformations included craniosynostosis (e.g. brachycephaly, turricephaly), midface hypoplasia, frontal bossing, pear-shaped nose, choanal stenosis/atresia, proptosis, dysplastic or low-set ears, radiohumeral or radioulnar synostosis, arachnodactyly, clinodactyly, camptodactyly, metacarpal/metatarsal synostoses, rocker-bottom feet, femoral bowing, joint contractures, and scoliosis (Supplementary Table 1). Only four patients had a very mild phenotype limited to difficulty bending the metacarpophalangeal joints.

PAI severity varied: 36% (31/87) required only occasional steroid replacement, whereas 20% (17/87) required lifelong glucocorticoid therapy. Treatment information was unavailable for the remaining patients.

Data on pubertal development were available for 53/136 patients. Of these, 33 were aged 9–19 years (27 females and 6 males), and 20 were adults (15 females and 5 males) (Supplementary Table 1). Delayed puberty was reported in 12 individuals (23%, 12/53). Six of these required hormonal induction and support for pubertal progression, all of whom were female; combined estrogen–progesterone therapy was most commonly used (9, 27, 28). In contrast, the two males with delayed puberty progressed spontaneously without medical intervention (9).

### Reported comorbidities and long-term organ system outcomes in patients with PORD

Of the 136 PORD patients identified, 72 (age range: neonatal to 48 years; 18 with a 46,XY karyotype and 54 with a 46,XX karyotype) had reported comorbidities and long-term outcomes. Patients were classified as having long-term outcomes if clinical manifestations occurred at or after pubertal age (>9 years). However, some features, such as delayed or advanced bone maturation, scoliosis, neurocognitive comorbidities, and ovarian cysts, have been reported as early as infancy. To ensure completeness and avoid systematic exclusion of clinically relevant phenotypes, patients presenting with these manifestations were also included in this section of the review.

The reported outcomes primarily affected the reproductive system (51/72, 71%), followed by the skeletal system (30/72, 42%), the cardiovascular system (9/72, 13%), and drug metabolism (1/72, 2%). Several patients (45/72, 63%) exhibited outcomes affecting more than one organ system.

Figure 1 summarizes the affected organ systems, associated comorbidities, and long-term outcomes observed in patients with PORD, with details provided below.

**Figure 1:**
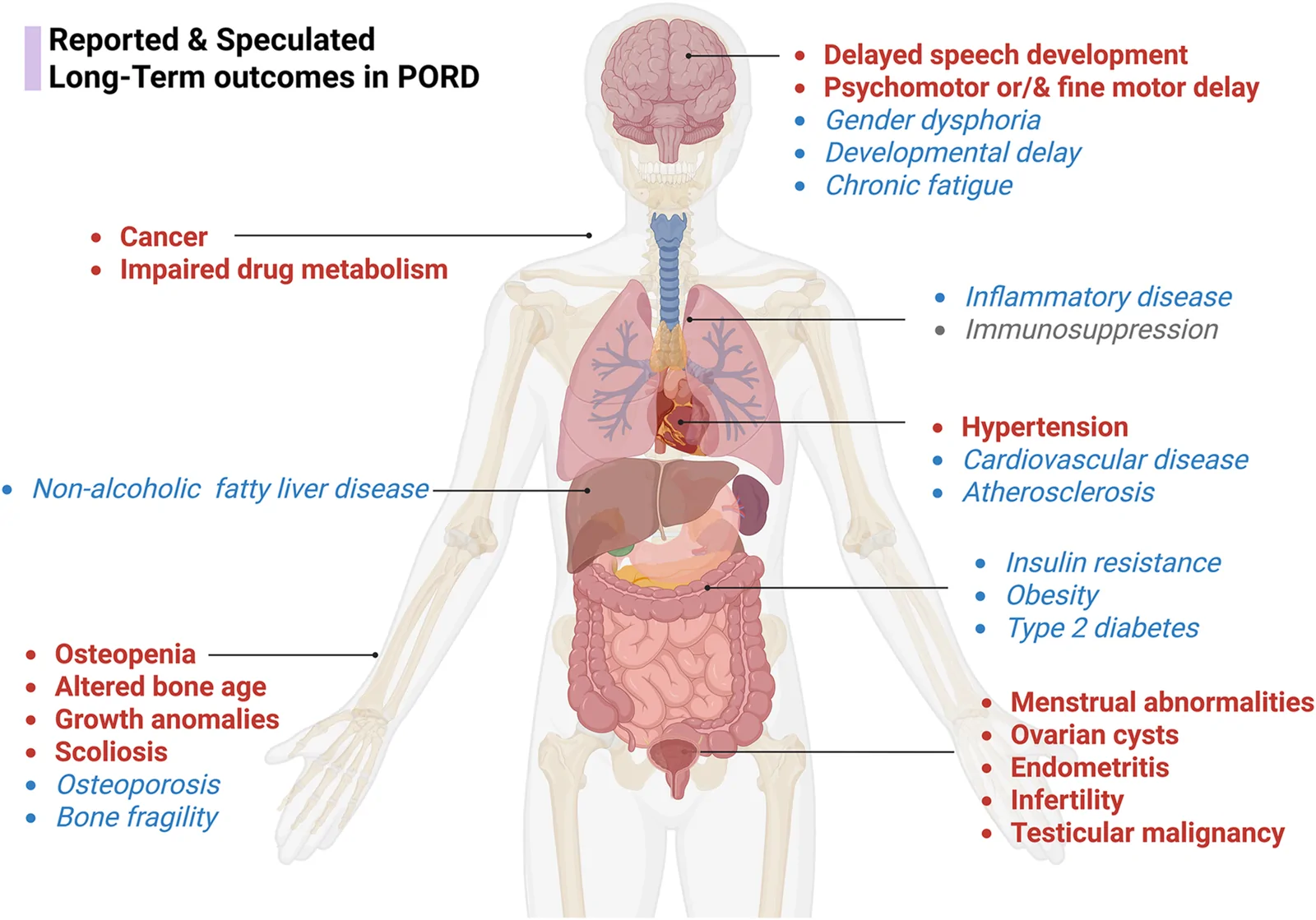
Reported and speculated comorbidities and long-term outcomes in patients with PORD. Terms in red indicate reported comorbidities and long-term outcomes. Terms in blue show hypothesized outcomes informed by POR-partner P450s. Italicized gray terms indicate potential side effects of glucocorticoid therapy. Created in BioRender*.*

#### PORD and the reproductive system

Infertility was a frequently reported long-term manifestation in both sexes. Among the 72 patients with long-term outcomes, 12 patients (10 females and two males), aged 24–47 years, were reported to be infertile (Supplementary Table 1). Of the 10 infertile females, seven also had menstrual abnormalities, including primary amenorrhea (4/10), secondary amenorrhea (1/10), and oligomenorrhea (2/10). Ovarian cysts were common in females with PORD, occurring in 36/72 (50%). Notably, no pregnancies resulting from natural conception have been reported to date, suggesting that assisted reproductive technologies are essential for fertility management in PORD. Indeed, six pregnancies have been achieved using various *in vitro* fertilization (IVF) protocols that involved controlled ovarian stimulation, hormonal substitution, glucocorticoid supplementation, and frozen embryo transfer and resulted in seven live births (6, 29, 30, 31, 32).

Menstrual disturbances were reported in 26 females (age range 12–38 years). Six (23%) had oligomenorrhea (6, 30, 33, 34), and 20 (77%) had amenorrhea. Among those with amenorrhea, 11 had primary amenorrhea (2, 6, 9, 31, 32, 34, 35, 36, 37), six had secondary amenorrhea (6, 34, 38), and in three cases the type was unspecified (29, 39, 40). Most were treated with hormone replacement therapy (HRT) containing estrogen and progesterone (Supplementary Table 1).

Ovarian cysts were reported in 36 female PORD patients, with onset ranging from 1 month to 38 years of age. These cysts are macrofollicular and differ from those seen in polycystic ovary syndrome (PCOS) (41). A major clinical concern is spontaneous cyst rupture, a potentially life-threatening event that may further compromise fertility (9).

Endometritis has been reported in three patients, one prepubertal (42) and two adults undergoing IVF (32). In the adult cases (ages 35 and 36 years), it was identified during hysteroscopy, which was performed before frozen embryo transfer, and both received anti-inflammatory treatment (32).

Male infertility has been documented in two cases (43, 44). In one, PORD was diagnosed during evaluation for infertility, and semen analysis revealed azoospermia (44). In the second case, serial semen analyses between ages 36 and 47 years consistently showed sperm parameters above the WHO lower fifth percentile, yet infertility was suspected because of the absence of conception with a proven fertile partner (43). A third patient was considered infertile based on oligoasthenoteratozoospermia (OAT); however, despite low sperm count, reduced motility, and abnormal morphology, this patient achieved natural conception at age 22 years (45). In addition, testicular biopsy in another case revealed reduced spermatogenesis, but fertility data were unavailable (46).

#### PORD and the skeletal system

Long-term skeletal findings included osteopenia, altered bone age, and growth abnormalities in 36 patients (Supplementary Table 1). One adult patient presented with spinal osteopenia (44); another patient showed low bone mineral density in both feet (47), and a 13-year-old girl exhibited significantly reduced bone mineral density (36). Progressive skeletal changes, such as scoliosis, or exacerbation of preexisting malformations – such as progressive restriction of elbow movement or worsening of multiple synostoses – were observed during growth in 18 individuals (2, 9, 26, 34, 35, 36, 40, 46, 47, 48, 49, 50, 51). Altered bone age, either delayed or advanced, was reported in 14 patients from 1 month to 36 years of age (Supplementary Table 1).

#### PORD and the cardiovascular system

Arterial hypertension was observed in both males (*n* = 3) (43, 44, 51) and females (*n* = 6) (2, 27, 51) with PORD, as well as in three pregnant patients (6, 30, 32) (Supplementary Table 1). Although there are no established guidelines for the management of hypertension in patients with PORD, standard antihypertensive medication was used in one case (43), while in others, dexamethasone was administered to lower blood pressure (2). Arterial hypertension has also been reported in children. Lee *et al.* (51) described two hypertensive patients, a girl and a boy aged 3 and 2 years, respectively; administration of glucocorticoids (e.g. hydrocortisone) led to normalization of their blood pressure. Thus, Dhir *et al.* (52) hypothesized that preferential CYP17A1 inhibition caused by the homozygous A287P POR variant (p.A284P; NM_001395413.1) may predispose to mineralocorticoid-mediated hypertension later in life, albeit milder than in isolated CYP17A1 deficiency; this was based on elevated corticosterone/cortisol ratios, and long-term confirmation remains pending.

In pregnant women with PORD, hypertension was observed at 12 weeks (30), 20 weeks (32), and 39 weeks of gestation, with the latter case complicated by preeclampsia requiring Caesarean section (6). One patient was treated with diltiazem (30), but no postpartum follow-up data were available.

#### PORD and tumors/cancer

One male adolescent presented with bilateral testicular adrenal rest tumors (TART) at the age of 14 years. Growth progression was controlled with daily hydrocortisone and dexamethasone therapy (45) (Supplementary Table 1). Although TART is associated with a risk of azoospermia and infertility, this patient successfully fathered a child. To date, no cases of ovarian adrenal rest tumors (OART) have been published. A separate case reported by Tomalik-Scharte *et al.* (27) described breast cancer at age 37 years in a woman with PORD, despite the absence of known risk factors, including a positive family history. The authors suggested that prolonged exposure to hormone replacement treatment (HRT) with estradiol (E2)/progestin may have contributed to tumor development.

#### PORD and neurocognition

Six of 72 patients (8%) exhibited signs of altered neurocognitive development. Delayed speech development was reported in four of these patients (35, 51, 53, 54) (Supplementary Table 1). They presented with symptoms consistent with delayed speech and showed articulation difficulties ranging from mild to severe, necessitating speech therapy. It must be emphasized that all exhibited an ABS phenotype. Moreover, three had unilateral or bilateral conductive hearing loss (35, 51, 53). One patient presented with delayed psychomotor and fine-motor development (53), and another patient was described as having an intellectual disability (35).

#### PORD and drug metabolism

POR is essential for CYP-dependent drug metabolism; however, research on drug metabolism in patients with PORD remains limited. Multiple *in vitro* and computational studies have shown that POR variants can impair the activity of drug-metabolizing partner CYP enzymes, potentially predisposing patients with PORD to drug toxicities and adverse drug reactions (17, 18, 19, 20, 21, 22, 23). The *in vivo* study by Tomalik-Scharte *et al.* (27) demonstrated impaired activity of five major drug-metabolizing CYP enzymes in a homozygous PORD patient and his carrier mother, both harboring the POR A287P variant (p.A284P; NM_001395413.1). Together with *in vitro* and computational data, these findings highlight the importance of evaluating drug metabolism in the context of specific POR mutations to minimize adverse reactions in patients with PORD.

### Phenotype–genotype correlation in reported patients with PORD

POR interacts with multiple microsomal P450 enzymes, and individual POR variants can differentially alter these interactions. This functional variability contributes to the broad phenotypic spectrum of PORD and likely underlies differences in comorbidities and long-term outcomes (1, 8, 23). Mapping available clinical outcome data (Supplementary Table S1) to specific POR variants indicates that, for more than half of the reported variants, adrenal involvement, atypical sex development, and skeletal manifestations are observed, whereas other organ systems are affected less frequently and may be variant specific (Fig. 2).

**Figure 2:**
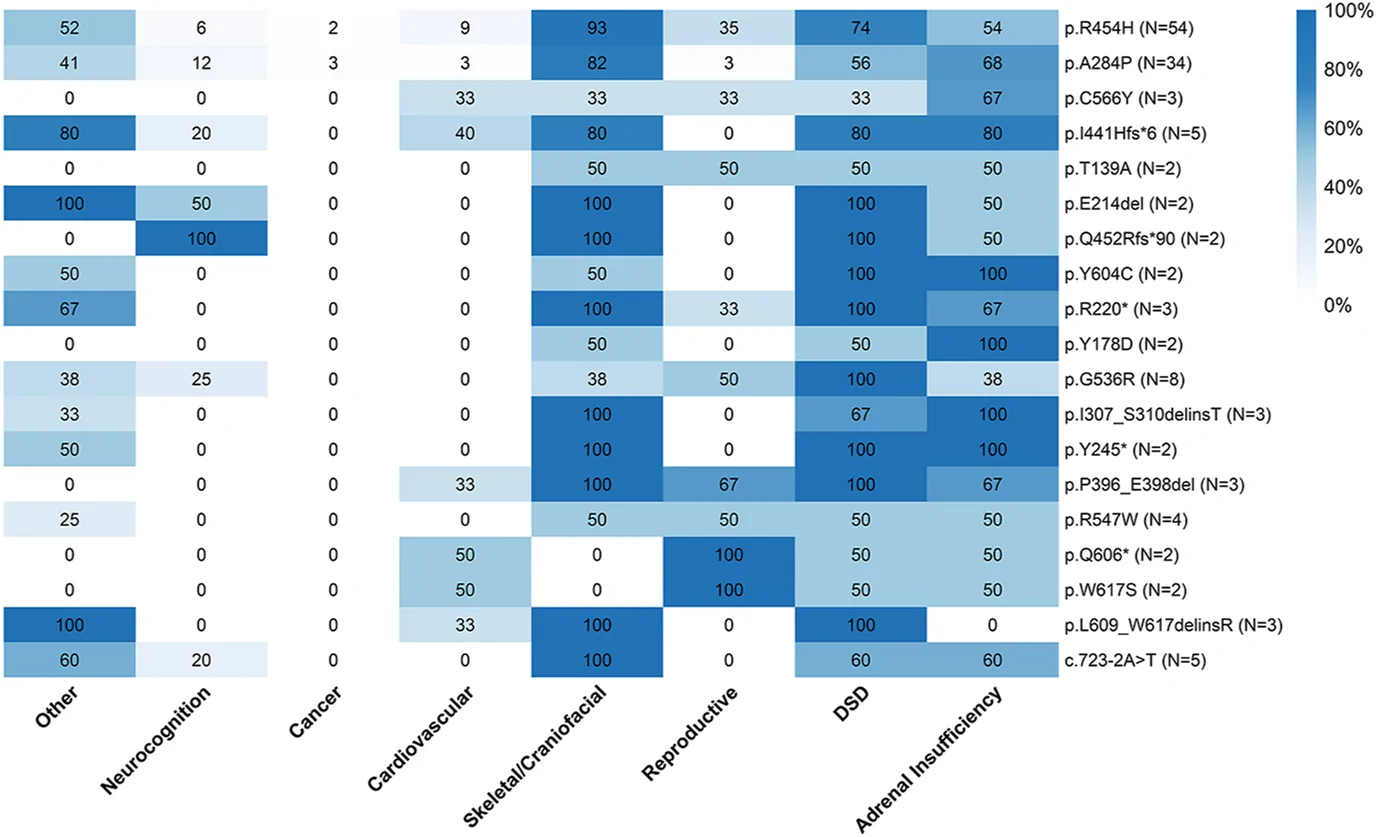
Phenotype matrix for all reported POR variants. The heatmap summarizes the clinical features observed in individuals carrying each reported POR variant. For each variant, N indicates the total number of affected individuals described in published case reports or clinical databases. The remaining columns list the number of individuals presenting with each phenotype category. Values represent raw counts of individuals with the indicated clinical manifestations; a single individual may contribute to multiple phenotype categories. Variants reported in only one individual are included here for completeness and to provide an overview of the full genotype–phenotype spectrum.

The two most prevalent POR variants, p.A284P (historically A287P) and p.R454H (historically R457H), show distinct genotype–phenotype patterns in the heatmap (Fig. 2) and Supplementary Table S1. p.A284P is consistently associated with a severe multisystem phenotype, including high rates of primary adrenal insufficiency, pronounced ABS-spectrum skeletal malformations, 46,XY undervirilization, 46,XX virilization, and recurrent ovarian cysts. Neurocognitive findings and hypertension also appear more frequently in this group, reflecting broad impairment across POR-dependent pathways. In contrast, p.R454H shows substantial but generally milder multisystem involvement. Individuals typically present with DSD, mild-to-moderate skeletal anomalies, and partial adrenal insufficiency, with fewer large-joint synostoses and less severe reproductive morbidity than p.A284P. Ovarian cysts, pubertal delay, and bone-age abnormalities occur but with greater variability. Together, these patterns suggest that p.A284P confers a high-penetrance, severe phenotype, whereas p.R454H retains more residual activity, resulting in more heterogeneous expressivity.

Overall, all these genotype–phenotype observations are subject to substantial limitations, including small sample sizes for most variants, reporting bias in available outcome data, and the young age and sex distribution of the included patients. Currently available longitudinal data remain limited and prevent definitive genotype-specific recommendations that could eventually inform risk-based surveillance strategies.

### Database-inferred network of POR partners and potential functional implications

Given POR’s central role as an electron donor for numerous enzymes involved in steroidogenesis and in the metabolism of drugs and xenobiotics, several potential health risks and long-term effects can be hypothesized for patients with PORD. By examining the physiological functions and known disease associations of individual POR-dependent enzymes (POR partners), it is plausible that POR variants, alone or in combination with variants or polymorphisms in partner enzymes, may impair their activity and lead to phenotypes resembling those observed in disorders linked to the partner enzymes themselves. Notably, the first biochemical characterization of PORD was inferred from decreased activities of steroid enzymes CYP21A2 and CYP17A1, causing a novel form of congenital adrenal hyperplasia termed ‘mixed oxidase disease’ (4).

Thus, the following section does not describe clinical findings in PORD patients. Instead, it synthesizes disease associations reported for POR-partner enzymes in the general population to highlight mechanistic vulnerabilities that may be relevant to PORD. These associations should be interpreted as biological hypotheses rather than established clinical phenotypes. Focusing on the 51 known POR partners, we reviewed the literature for disease associations and identified numerous studies describing the effects of polymorphisms or variants in partners across multiple organ systems. A summary of this evidence is shown in Fig. 3 and included in Fig. 1 (items in blue).

**Figure 3:**
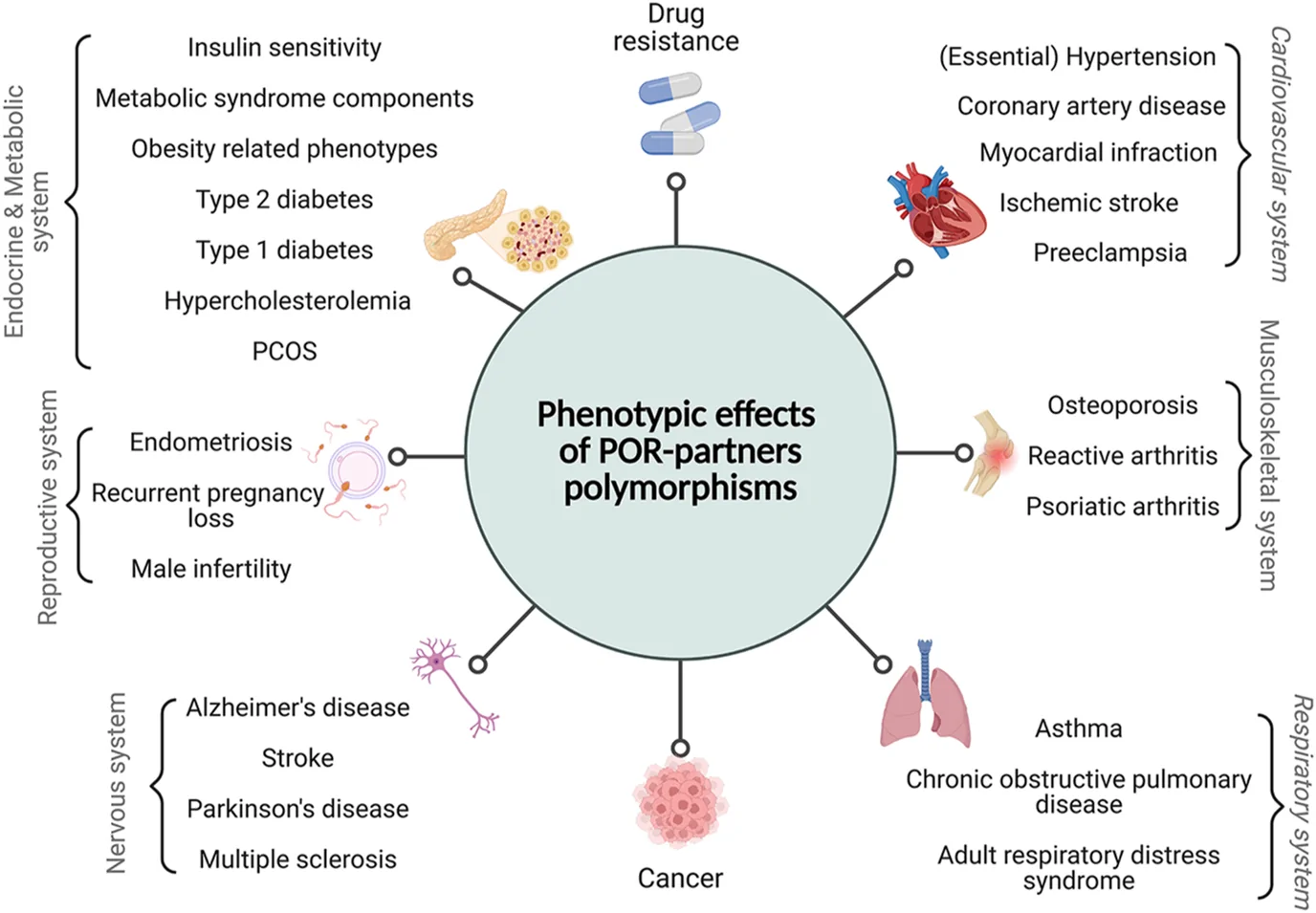
Summary of reported phenotypic effects across organ systems of polymorphisms/variants in POR-partner proteins. PCOS, polycystic ovary syndrome. Created in BioRender*.*

### Phenotypes associated with polymorphisms of POR partners and speculated health risks

In the following section, phenotype associations reported for POR partners are summarized according to the related organ systems. Source data are provided in Supplementary Table S2.

#### Polymorphisms in POR partners and cancer

Of the 51 known POR partners, 22 (*CYP1A1, CYP1A2, CYP1B1, CYP2A13, CYP2B6, CYP2C8, CYP2C9, CYP2C19, CYP2D6, CYP2E1, CYP2W1, CYP3A4, CYP3A5, CYP3A43, CYP4B1, CYP4F2, CYP4F12, CYP17A1, CYP19A1, CYP26B1, CYB5A, *and* HMOX1*) have been reported to harbor polymorphisms associated with various cancers (Supplementary Table 2). These associations were derived from clinical case–control studies and meta-analyses across diverse populations, with detailed references provided in the Supplementary Table 2. The most frequently reported cancer associations included breast cancer (*CYP1A1*, *CYP1A2, CYP1B1, CYP2C19, CYP2D6, CYP4B1, CYP17A1, CYP19A1, *and* CYB5A*), prostate cancer *(CYP1A1, CYP1B1, CYP2D6, CYP3A4, CYP3A5, CYP3A43, CYP17A1, *and* CYP19A1),* and lung cancer *(CYP1A1, CYP1B1, CYP2A13, CYP3A4, CYP4B1, CYP4F2, *and* CYP19A1).* These associations reflect the roles of these enzymes in drug and xenobiotic metabolism, as well as in hormone synthesis and regulation, which can influence susceptibility to hormone-sensitive cancers.

#### Polymorphisms in POR partners and the cardiovascular system

POR-partner enzymes also influence cardiovascular health by modulating the metabolism of endogenous steroids, fatty acids, and lipids, as well as by contributing to oxidative stress and endothelial dysfunction (55, 56, 57). Sixteen of the 51 POR partners have been linked to mild to severe cardiovascular diseases (*CYP1A1, CYP2C9, CYP2J2, CYP2R1, CYP3A4, CYP3A5, CYP4A11, CYP2C19, CYP4A22, CYP4F2, CYP4V2, CYP7A1, CYP17A1, CYP19A1, CYP7B1, *and* HMOX1)* (Supplementary Table 2). Polymorphisms in 11 POR partners (*CYP1A1, CYP2C9, CYP2R1, CYP3A4, CYP3A5, CYP4A11, CYP2C19, CYP4V2, CYP7A1, CYP17A1, *and* HMOX1*) have been associated with hypertension or essential hypertension. Increased risk of coronary artery disease has been linked to polymorphisms in 12 POR partners (CYP1A1, CYP2J2, CYP2R1, CYP3A4, CYP4A11, CYP4A22, CYP4F2, CYP4V2, CYP7A1, CYP17A1, CYP19A1, and CYP7B1). Polymorphisms in *CYP2C19, CYP4F2, CYP4V2, CYP2J2, *and* CYP2R1* have been associated with a higher incidence of ischemic stroke, myocardial infarction, or both (*CYP2J2, CYP2R1, CYP3A5, CYP2C19, *and* CYP4F2*, Supplementary Table 2).

#### Polymorphisms in POR partners and the endocrine and metabolic systems

Genetic polymorphisms in POR partners can disrupt steroidogenesis, as well as fatty acid, cholesterol, and bile acid metabolism, with implications for endocrine and metabolic health (58, 59). Polymorphisms in 13 POR partners (*CYP1A1, CYP1B1, CYP2E1, CYP2C8, CYP2C9, CYP2R1, CYP2C19, CYP3A4, CYP7A1, CYP8B1, CYP17A1, CYP19A1, *and* HMOX1*) have been linked to metabolic and endocrine disorders. Type 2 diabetes was among the most frequently reported associations, involving variants in six partners (*CYP1B1, CYP2E1, CYP2C9, CYP2C19, CYP7A1, *and* CYP17A1*) (Supplementary Table 2). Additional associations include PCOS (*CYP1A1, CYP17A1, *and* CYP19A1*), components of the metabolic syndrome (*CYP2R1 *and* HMOX1*), insulin sensitivity (CYP8B1), hypercholesterolemia (*CYP7A1*), obesity-related phenotypes (CYP19A1), and type 1 diabetes (*CYP2R1*) (Supplementary Table 2).

#### Other implications seen with polymorphisms in POR partners

Additional disease associations involving POR-partner enzymes have been reported across multiple organ systems. In the respiratory system, chronic obstructive pulmonary disease (COPD) has been linked to polymorphisms in seven POR partners (*CYP1A1, CYP1A2, CYP2B6, CYP3A4, CYP4F2, *and* HMOX1;* Supplementary Table 2). In the reproductive system, endometriosis has been linked to genetic variations in five POR partners (*CYP1A1, CYP2C19, CYP19A1, CYP17A1, *and* HMOX1*), while recurrent pregnancy loss and male infertility have been linked to *CYP1A1* polymorphisms (Supplementary Table 2). Within the nervous system, Alzheimer’s disease has been associated with three POR partners (*CYP17A1, CYP19A1, *and* CYP46A1*), Parkinson’s disease with *CYP2D6*, and multiple sclerosis with *CYP2R1* polymorphisms (Supplementary Table 2). In the skeletal system, polymorphisms in *CYP1B1, CYP17A1, *and* CYP19A1* have been associated with osteoporosis, particularly in postmenopausal women, while *CYP1A1* variants have been implicated in reactive or psoriatic arthritis (Supplementary Table 2, including all references).

### Specific disorders associated with pathogenic variants in POR partners

In addition to the effects of POR-partner polymorphisms, we searched the online Mendelian inheritance in man (OMIM) database for gene–phenotype associations. Fifteen of the 51 POR-partner genes were linked to specific monogenic diseases (Table 1). Although such severe disorders are not observed in patients with PORD, these associations highlight the types of anomalies and organ systems that may be influenced by dysfunction of POR-dependent enzymes, helping to contextualize the broad phenotypic spectrum seen in PORD.

**Table 1:** Landscape of POR partners’ disease associations from online Mendelian inheritance in man (OMIM).

POR-partner/phenotype	OMIM number	Inheritance
CYP1B1		
Anterior segment dysgenesis 6, multiple subtypes	617315	Autosomal recessive
Glaucoma 3A, primary open angle, congenital, juvenile, or adult onset	231300	Autosomal recessive
CYP2A6		
Coumarin resistance (also known as warfarin resistance)	122700	Autosomal dominant
CYP2B6		
{Efavirenz central nervous system toxicity, susceptibility to}c and efavirenz, poor metabolism of	614546	
CYP2C8		
{Drug metabolism, altered, CYP2C8-related}	618018	
CYP2C9		
Tolbutamide poor metabolizer	601130	
Warfarin sensitivity	122700	Autosomal dominant
CYP2C19	124020	Autosomal recessive
Clopidogrel, impaired responsiveness to		
Mephenytoin poor metabolizer		
Omeprazole poor metabolizer		
Proguanil poor metabolizer		
CYP2D6	608902	Autosomal recessive
{Debrisoquine sensitivity}		
{Codeine sensitivity}		
CYP2R1		
Rickets due to defect in vitamin D 25-hydroxylation deficiency	608713	Autosomal recessive
CYP2U1		
Spastic paraplegia 56, autosomal recessive	610670	Autosomal recessive
CYP3A4		
Vitamin D-dependent rickets, type 3	124010	Autosomal dominant
CYP3A5		
{Hypertension, salt-sensitive essential, susceptibility to}	605325	Multifactorial
CYP4F22		
Ichthyosis, congenital, autosomal recessive 5	611495	Autosomal recessive
CYP4V2		
Bietti crystalline corneoretinal dystrophy (also known as Bietti crystalline dystrophy)	608614	Autosomal recessive
CYP7B1		
Bile acid synthesis defect, congenital, 3	613812	Autosomal recessive
Spastic paraplegia 5A, autosomal recessive	270800	Autosomal recessive
CYP17A1	609300	Autosomal recessive
17-Alpha-hydroxylase/17,20-lyase deficiency		
17,20-Lyase deficiency, isolated		
CYP19A1		
Aromatase deficiency	613546	
CYP21A2	201910	Autosomal recessive
Adrenal hyperplasia, congenital, due to 21-hydroxylase deficiency		
Hyperandrogenism, nonclassic type, due to 21-hydroxylase deficiency		
CYP26B1		
Craniosynostosis with radiohumeral fusions and other skeletal and craniofacial anomalies	614416	Autosomal recessive
CYP26C1	608428	Autosomal recessive
Focal facial dermal dysplasia 4 (FFDD type IV)		
CYB5A		
Methemoglobinemia and ambiguous genitalia	250790	
HMOX1		
{Pulmonary disease, chronic obstructive, susceptibility to}	606963	
Heme oxygenase-1 deficiency	614034	Autosomal recessive

## Gaps and future directions

Despite increasing recognition of PORD as a multisystem disorder, evidence on long-term outcomes remains sparse. Early research has understandably focused on severe neonatal and childhood manifestations, particularly endocrine dysfunction, DSD, and skeletal malformations, while age-dependent or subtler consequences have been largely overlooked. As a result, there are currently no evidence-based guidelines for lifelong management. The findings of this review highlight several areas where systematic investigation is still needed.

Hypertension has been documented across all age groups in PORD and is biologically plausible given the involvement of multiple POR-dependent enzymes in vascular regulation. The pathophysiology of hypertension in PORD likely reflects a combination of mineralocorticoid-mediated and endothelial mechanisms. Biochemical observations in affected patients, including suppressed renin activity and altered mineralocorticoid profiles, support the hypothesis that impaired CYP17A1 activity may shift steroidogenesis toward corticosterone production and thereby increase mineralocorticoid tone (1, 8, 60). Beyond these classical pathways, POR also appears to modulate endothelial vasoregulation. Endothelial-specific POR knockout mice exhibit markedly reduced synthesis of key vasodilators (e.g. epoxyeicosatrienoic acids) and a concomitant diversion of arachidonic acid metabolism toward COX-derived vasoconstrictor prostanoids, resulting in endothelial dysfunction and hypertension (61, 62). Consistent with these findings, a recent study in ten patients with PORD demonstrated elevated blood pressure, microvascular abnormalities, and increased circulating thromboxane-related vasoactive mediators, suggesting enhanced COX-mediated vasoconstrictor tone in humans as well (57). Despite these mechanistic insights, hypertension is not a universal feature of PORD. Genotype–phenotype variability, including variant-specific residual enzyme activity, likely contributes to differences in mineralocorticoid excess and endothelial impairment across individuals. Moreover, genetic polymorphisms in POR-interacting partner enzymes may further modify susceptibility to hypertension. Larger cohorts and functional studies will be required to determine whether specific POR variants or partner-gene polymorphisms predispose to more pronounced cardiovascular involvement. However, because untreated hypertension carries substantial long-term risks, including renal, ocular, and cardiac complications (63, 64), lifelong blood pressure monitoring should be incorporated into routine care, with management aligned with established clinical guidelines and escalation of diagnostic evaluation when abnormalities arise.

POR is indispensable for the activity of major drug-metabolizing CYP enzymes (65), and although *in vivo* data remain limited, impaired POR function may alter drug clearance, increase susceptibility to adverse reactions, or reduce therapeutic efficacy. Experimental models further suggest vulnerability to hepatocellular injury (66), lipid accumulation (67), and progression toward nonalcoholic fatty liver disease (NAFLD) (68) and fibrosis (59). These observations underscore the need for pharmacovigilance, personalized dosing strategies, and routine assessment of liver function in PORD.

Disruption of bile acid synthesis and cholesterol regulation through POR-dependent enzymes may contribute to dyslipidemia, cholestasis, or proatherogenic lipid profiles (56, 58, 69). Although such conditions have not yet been reported clinically in PORD, likely because of their age-dependent nature, longitudinal monitoring of lipid metabolism is warranted as patients reach adulthood and older age.

Several POR-partner enzymes participate in eicosanoid metabolism, influencing inflammatory and immune responses. Disruption of these pathways may predispose to chronic inflammation or immune dysregulation. While these outcomes have not been systematically evaluated in PORD, associations between POR-partner variants and inflammatory conditions in the general population highlight the need for targeted research into immune function in PORD (41, 70).

Infertility is one of the most clinically significant long-term outcomes in PORD. The absence of reported spontaneous pregnancies in women underscores the importance of early reproductive counseling and timely referral to fertility specialists. In males, early assessment of gonadal function and semen parameters may guide reproductive planning. Prospective studies are needed to determine the feasibility of natural conception and to optimize assisted reproductive protocols (32).

POR-dependent disruption of vitamin D metabolism may contribute to long-term skeletal complications beyond congenital malformations (58). Monitoring of vitamin D status, bone mineral density, and growth parameters should therefore be part of routine follow-up, particularly during adolescence and adulthood.

Associations between POR-partner variants and neurodegenerative diseases raise the possibility that POR dysfunction may influence long-term neurological health. Although no such outcomes have been reported in PORD, this represents an important area for future investigation.

Long-term glucocorticoid treatment, frequently required in PORD, carries well-known risks, including metabolic disturbances, hypertension, osteoporosis, and psychiatric or ophthalmological complications (71). These treatment-related effects must be considered when interpreting long-term outcomes and designing follow-up protocols.

No studies have evaluated quality of life, psychosocial well-being, or gender-related outcomes in PORD. Given the high prevalence of DSD and the potential for atypical pubertal development, structured psychosocial assessment should be integrated into long-term care. This represents a major and currently unaddressed gap (41, 72).

The broad range of potential manifestations reflects the central role of POR as the obligate electron donor to numerous CYP and non-CYP enzymes. Although many mechanistic links remain hypothetical, they provide a biologically coherent framework for understanding the multisystem nature of PORD. Targeted longitudinal studies, mechanistic investigations, and international registry efforts will be essential to define the true long-term phenotype and to develop evidence-based management guidelines.

A coordinated international infrastructure is now essential to overcome the fragmented and predominantly pediatric nature of current PORD evidence. Establishing prospective longitudinal cohorts and a dedicated international registry would allow systematic capture of endocrine, skeletal, reproductive, cardiovascular, neurocognitive, hepatic, and psychosocial outcomes across the lifespan. Such efforts would enable robust variant-specific risk stratification, clarify age-dependent manifestations, and support evaluation of treatment effects. Ultimately, these data are required to develop evidence-based surveillance algorithms and harmonized long-term management protocols for individuals with PORD.

## Conclusion

This review underscores that PORD is a complex multisystem disorder for which long-term consequences remain insufficiently characterized. While early clinical features such as adrenal insufficiency, DSD, and skeletal malformations are well documented, far less is known about the evolving health needs of adolescents and adults. Emerging evidence, together with insights from POR-partner biology, suggests that individuals with PORD may be at risk for cardiovascular, metabolic, hepatic, reproductive, skeletal, and potentially neurological complications (Box 1). However, the scarcity of longitudinal data limits our ability to anticipate, prevent, or manage these outcomes effectively. There is a clear need for coordinated international efforts, including prospective cohort studies, mechanistic research, and the development of standardized follow-up protocols. A deeper understanding of POR-dependent pathways will not only refine clinical care for patients with PORD but also illuminate broader principles of human metabolism, endocrine regulation, and CYP-mediated physiology.

**Box 1:** Key messages

**Confirmed clinical profile and long-term findings in PORD** **PORD presents with a characteristic multisystem profile**, most commonly combining adrenal insufficiency, differences of sex development, and Antley–Bixler-like skeletal malformations. **Comorbidities affect multiple organ systems**, including reproductive, skeletal, cardiovascular, neurocognitive, and drug-metabolism pathways. **Long-term outcomes remain insufficiently defined**, and no evidence-based lifelong management guidelines currently exist. **Hypertension occurs in a subset of patients** across age groups and requires systematic monitoring to prevent renal, ocular, and cardiovascular complications. **POR dysfunction can impair drug metabolism** and increase susceptibility to adverse drug reactions, highlighting the need for pharmacovigilance and liver monitoring. **Infertility is a major long-term concern** in both sexes, making early reproductive counseling and evaluation essential. **Bone health may be compromised**, supporting routine monitoring of bone density and vitamin D status. **Neurocognitive findings have been reported** in a minority of individuals, particularly those with pronounced ABS phenotypes. **Chronic glucocorticoid therapy may contribute to morbidity** and should be considered when interpreting long-term outcomes. **Psychosocial and gender-related well-being remain unstudied** despite high DSD prevalence, representing a critical unmet need.

**Suspected or theoretical risks based on POR-partner biology** **Disruption of bile-acid and lipid metabolism may predispose** to dyslipidemia and proatherogenic profiles, warranting longitudinal metabolic assessment. **Immune and inflammatory dysregulation is biologically plausible** through POR-dependent eicosanoid pathways but remains unexplored in PORD. **Possible neurological implications of POR dysfunction** have not been investigated and represent an important research gap. **Additional endocrine and reproductive vulnerabilities** may arise from polymorphisms in POR-partner enzymes, although these have not been observed in PORD patients. **Skeletal risks such as osteoporosis or inflammatory arthropathies** are suggested by partner–gene associations but remain theoretical in the context of PORD.

## Supplementary materials



## Declaration of interest

The authors declare that there is no conflict of interest that could be perceived as prejudicing the impartiality of the work reported.

## Funding

This work was supported by the Swiss National Science Foundation grant DSDgene (3200-0-239909).

## Author contribution statement

CK and AT performed the data search, participated in scientific discussions, and wrote the first draft. CEF performed the data search, participated in scientific discussions, designed the paper, and reviewed the final manuscript.

## Data availability

All data discussed in this review are derived from previously published studies, which are cited in the reference list.

## Ethics statement

As this work is a literature review based solely on previously published, anonymized data, without any new data collection or involvement of human participants, ethical approval was not necessary.
