# Supplementary material for: Long-term health outcomes and comorbidities in cytochrome P450 oxidoreductase (POR) deficiency: a scoping review of evidence and knowledge gaps
Source: Endocr Connect. 2026 Oct 15;15(10):e260354. doi: 10.1530/EC-26-0354 (PMC13695394; doi:10.1530/EC-26-0354)
Supplement: Supplementary file 1 [file EC-26-0354_supplementary_materials.pdf]

Supplementary Material for:

Long-term health outcomes and comorbidities in cytochrome P450 oxidoreductase (POR) deficiency: a scoping review of current evidence and knowledge gaps

Table of Contents

Table S 1: Clinical characteristics of patients with PORD. 2

Table S 2: Phenotypes of polymorphisms in POR-partner genes.......... 21

References.......... 29

Figure S1: Selection process of the scoping review.......... 42

PRISMA checklist.......... 43

Table S 1: Clinical characteristics of patients with PORD.

Ref	Current age/age at referral (r)	Karyo-type	POR Variant (as reported in the publication)	POR Variant (HG, NM_001395413.1.)	Adrenal function	DSD at birth	Pubertal development: Tanner stages, G (Genitals), B (Breast), P/PH (Pubic hair), A (Axillary hair), M (Menarche)	Reproductive health	Bone score, Skeletal features	Others
Flück et al. 2004 (1)	Neonatal (r)	46, XX	p.R457H/ c.731+1G→A	p.R454H/ c.722+1G>A	AI	clitoromegaly, labial fusion, single urogenital orifice	-		7 M, craniosynostosis, severe midface hypoplasia, large joint synostosis, hand/feet malformations	maternal virilization (20 weeks)
"	Neonatal (r)	46, XY	p.V492E/-	p.V489E/ p.A284P	AI?	testes, small, <1 ml, in labioscrotal folds, 8 mm phallus, urogenital sinus	-		8 M, midface hypoplasia, craniosynostosis, radioulnar malformation, one hand malformation, narrow upper thoracic cage, tall stature, scoliosis	mother with ABS features
"	Neonatal (r)	46, XY	p.A287P/ p.A287P	p.A284P/ p.A284P	abnormal	micropenis, 3rd degree hypospadias, labioscrotal fusion, chordee	-		10 S, complicated midface hypoplasia, severe craniosynostosis, hand/feet malformation, radiohumeral synostosis, femoral bowing	oligohydramnios, atrial septal defect, significant respiratory complications incl.apnea at neonatal age. Seizures were suspected, EEG showed right temporal spike wave discharges. Growth on the low percentiles. Strawberry haemangioma at frontal hairline and nuchal naevus flammeus
"	23 y (r)	46, XX	p.C569Y/ p.V608F	p.C566Y/ p.V605F	disordered steroidogenesis, mild arterial hypertension	-	normal breast development	primary amenorrhea, hypoplastic uterus, bilaterally enlarged cystic ovaries	0, no	

Adachi et al. 2004 (2)	1y (r)	46, XY	c.1329insC/p. R454H	p.I441Hfs*6/ p.R454H	AI	-	-	-	7 M, moderate craniosynostosis, midface hypoplasia, radiohumeral synostosis, hand/feet malformations	cardiac arrest due to upper airway obstruction twice during infancy, hydrocephalus
“	11y (r)	46, XX	c.1698insC/p. R454H	p.Y564Ls*8/ p.R454H	biochemic ally insufficient , no clinical signs	clitoromegaly, labia minora fusion, hypoplastic labia majora, hypoplastic vagina/uterus	delayed puberty = no signs at 11y	-	5 M, severe midface hypoplasia, moderate craniosynostosis, bilateral radiohumeral synostosis	maternal virilization
Wudy et al. 2004 (3)	Neonatal (r)	46, XY	p.A287P/ p.A287P	p.A284P/ p.A284P	AI, slightly elevated potassium (5.4-5.52)	-	-	-	4 MI, moderate midface hypoplasia, severe craniosynostosis, one hand malformation	umbilical hernia, slight retardation (walk free at 15 months, speak few words since 18 months). Mother had HELLP at 34 weeks of gestation
Huang et al. 2005 (4)		46, XY	p.F646del/ p.A287P	p.F643del/ p.A284P	abnormal	micropenis, hooded prepuce, hypospadias, chordee, anteriorly placed anus	-	-	12 S, complicated midface hypoplasia, severe craniosynostosis, hand/feet malformations, one large joint synostosis, hemivertebrae, bronchomalacia, absent auditory meatus and ossicular chain on one side	horseshoe kidney
“		46, XY	p.A287P/ Frame 2	p.A284P/ Frame 2	abnormal	micropenis, hypospadias, bilateral cryptorchidism	-	-	6 M, moderate midface hypoplasia, craniosynostosis, radiohumeroulnar synostosis, one hand malformation,	hypertelorism, conductive hearing loss, died at age of 3 y
		46, XY	p.Q153R/ p.Q153R	p.Q150R/ p.Q150R	-	normal external genitalia	-	-	5 M, craniosynostosis, one large joint synostosis, femoral bowing, one hand malformation	abortion at 22 weeks
		male	p.R457H/ p.R457H	p.R454H/ p.R454H	-	scrotal hypoplasia, small penis	-	-	8 M, complicated midface hypoplasia, moderate craniosynostosis, one large joint synostosis, femoral bowing, one hand malformation	Arnold-Chiari malformation
		male	p.R457H/p.Y459H	p.R454H/ p.Y604H	-	undescended testes	-	-	9 S, complicated midface hypoplasia, moderate craniosynostosis, one hand malformation, one large	

									joint synostosis, complicated femoral bowing	
		46, XX	p.A287P/ Frame 3	p.A284P/ Frame 3	-	clitoromegaly, no vaginal orifice, vesicovaginal fistula	-	-	10 S, complicated midface hypoplasia, moderate craniosynostosis, hand/feet malformations, one large joint synostosis, femoral bowing	small for gestational age
		46, XX	p.A287P/ p.A287P	p.A284P/ p.A284P	-	-	-	-	8 M, severe midface hypoplasia, moderate craniosynostosis, hand/feet malformations, one large joint synostosis, femoral bowing	
		46, XX	p.A287P/ Frame 4	p.A284P/ Frame 4	abnormal		-	-	4 MI, craniosynostosis, one large joint synostosis, femoral bowing	
		46, XX	p.R457H/ Frame 6	p.R454H/ Frame 6	abnormal	urogenital sinus, vestigial uterus, posteriorly placed septated vagina.	-	-	4 MI, moderate craniosynostosis, one large joint synostosis, femoral bowing	
		46, XY	p.A287P/ Frame 7	p.A284P/ Frame 7	abnormal	-	-	-	3 MI, severe midface hypoplasia, moderate craniosynostosis	
		46, XY	p.L565P/ p.R457H	p.L562P/ p.R454H	abnormal	ambiguous genitalia, microphallus	-	-	7 M, complicated midface hypoplasia, moderate craniosynostosis, one large joint synostosis, femoral bowing	
		46, XY	p.T142A/ Frame 5	p.T139A/ Frame 5	abnormal	microphallus, bifid scrotum, glandular hypospadias	spontaneous puberty	-	-	
Fukami et al. 2005 (5)	26 y	46, XY	p.R457H /p.L612_W620 delinsR	p.R454H/ p.L609_W61 7delinsR	-	micropenis, cryptorchidism, hypospadias	P2		6 M, moderate craniosynostosis, midface hypoplasia, elbow extension deficit, hand malformations, scoliosis (13y),	conduction deafness, R-vesicourethral reflux, delayed bone age
	29 y	46, XY	p.R457H /p.L612_W620 delinsR	p.R454H/ p.L609_W61 7delinsR	-	micropenis, cryptorchidism	P3		moderate craniosynostosis, midface hypoplasia, radiohumeral synostosis, hand malformations,	conduction deafness
	17.5 y	46, XY	(G5G)c /p.R457H	p.G2= /p.R454H	-	cryptorchidism	P3		5 M, moderate craniosynostosis, midface hypoplasia, elbow	delayed bone age

									extension deficit, hand malformations	
	2 y	46,XX	p.R457H/ p.R457H	p.R454H/ p.R454H	-	clitoromegaly, complete labial fusion	B1, P1		5 M, moderate craniosynostosis, midface hypoplasia, elbow extension deficit, hand/feet malformations	maternal virilization, advanced bone age
	9.5 y	46, XX	p.R457H/ p.R457H	p.R454H/ p.R454H	-	clitoromegaly, complete labial fusion	Breast = Budding, P1		moderate craniosynostosis, midface hypoplasia, elbow extension deficit, hand/feet malformations	
	15 y	46, XX	p.R457H/ p.R457H	p.R454H/ p.R454H		clitoromegaly	B2, P2	ovarian cyst	3 MI, moderate craniosynostosis, hand malformations	maternal virilization, delayed bone age, B-vesicourethral reflux
Fukami et al. 2006 (6)	4,5 y	46, XY	p.R457H/ p.R457H	p.R454H/ p.R454H	AI	Imperforate anus	-	-	-	no maternal virilization
	3 months (r)	46, XX	p.R457H/ p.R457H	p.R454H/ p.R454H	-	severely virilized external genitalia (Prader V) with a phallus-like structure of 1.0 cm length and complete labial fusion, bilateral polycystic ovaries (at age 3 m)	-	-	1 MI, one feet malformation	maternal virilization and low estriol in the 3rd trimester
	2 months (r)	46, XX	p.R457H/ p.R457H	p.R454H/ p.R454H	-	severely virilized external genitalia (Prader stage 5) with a phallus-like structure of 2.0 cm length and complete labial fusion, bilateral polycystic ovaries (at age 2 m)	-	-	3 MI, moderate craniosynostosis, hand malformations	maternal virilization in third trimester
Williamson et al. 2006 (7)	Neonatal (r)	46, XX	p.A287P/ p.H604P	p.R454H/ p.H601P	AI	clitoromegaly (regressed), complete labial fusion (Prader III-IV)	-	-	8 M, severe midface hypoplasia, moderate craniosynostosis, hand/feet malformation, elbow extension deficit, advanced bone age at 5 weeks (BA = 6 months), lumbar lordosis (13 months) decreased by 39 months, delayed ossification of the femoral epiphyses and malformed	excess nuchal skin, bilateral mild to moderate conductive hearing loss at 4y, delayed dental eruption (born at 36 weeks of gestation by C-section), VUR (4 months), 2/6 systolic murmur, sleep apnea, hyperopia, GERD (24

									calvarium, by 4y bone age = chronological age	months) and then chronic constipation (5y), cyclic vomiting syndrome (9y), mild delay in fine motor skills, speech therapy after 2y
Hershkovitz et al. 2008 (8)	21y	46, XY	p.G539R/ p.G539R	p.G536R/ p.G536R	AI	micropenis, inguinal testes, underdeveloped scrotum	normal puberty onset, sertoli cell failure	Tanner 5 (21y)	0	consanguineous parents, Patient 1
	Neonatal (r)	46, XY	p.G539R/ p.G539R	p.G536R/ p.G536R	AI	micropenis, chordee, scrotal hypospadias, and small testes (0.5ml), bifid scrotum	retained one dysfunctional testis, a small penis, sertoli cell failure, Tanner 2 (14.6y)	-	0	Patient 1's younger brother, consanguineous parents
	10 months (r)	46, XY	p.G539R/ p.G539R	p.G536R/ p.G536R	AI	micropenis	normal onset puberty, Tanner 5 (16.5y)	-	0	consanguineous parents
	1 d (r)	46, XY	p.G539R/ p.G539R	p.G536R/ p.G536R	AI	micropenis with chordee, undescended testes	undervirilized	-	0	consanguineous parents
Sahakitrungruang et al. 2009 (9)	Neonatal (r)	46, XY	p.delE217/ p.A287P	p.E214del/ p.A284P	AI	micropenis, chordee	-	-	5 M, moderate midface hypoplasia, severe craniosynostosis, radiohumeral synostosis	poor speech articulation (3y), partial hearing loss (right side)
	18 y (r)	46, XX	p.N185K/ p.L577R	p.N182K/ p.L574R	AI	-	normal breast development	Primary amenorrhea, infertility (24y)	0	-
	Neonatal (r)	46, XX	p.G539R/c.1363delC	p.G536R/ p.Q452Rfs*90	AI, elevated aldosterone	ambiguous genitalia	no breast development, no pubic hair, prepubertal uterus, ovaries not seen	-	4 M, craniosynostosis, radiohumeral synostosis, severe scoliosis, delayed bone age (10y) (at 16 11/12 y)	significant intellectual disability
	Neonatal (r)	46, XX	p.G539R/c.697–698insGAAC	p.G536R/ p.V233Gfs*46	AI, hyponatremia, elevated renin and aldosterone	phallus (1.5 x 0.7 cm) with perineal hypospadias, fused labioscrotal folds, no palpable gonads	large ovarian cyst (4 months)	-	4 MI, severe midface hypoplasia, severe craniosynostosis	divergent strabismus, delayed speech development
Ko et al. 2009 (10)	7 months	46, XX	p.I444fsX449/ p.R457H	p.I441Hfs*6/ p.R454H	AI; normal levels of 17-OHP in	partial labial fusion, urogenital sinus	-	-	7 M, severe midface hypoplasia, moderate craniosynostosis, hand	low maternal unconjugated estriol (serum) during 2nd

					neonatal screening				malformation, radiohumeral synostosis, femoral bowing (left): benign enlargement of the subarachnoid space	trimester of pregnancy, child developed grunting sound during respiration shortly after birth, was treated in NICU for 7d. Narrow nasopharynx
Iijima et al. 2009 (11)	Neonatal (r)	46, XX	p.348delV/ p.R457H	p.V345del/ p.R454H	AI	imperforate anus with anovestibular fistula	-	-	4 MI, moderate midface hypoplasia, moderate craniosynostosis, one hand malformation, radioulnar synostosis	respiratory distress and stridor as newborn
Tomalik-Scharte et al. 2010 (12)	48 y	46, XX	p.A287P/ p.A287P	p.A284P/ p.A284P	AI, hypertension	severely virilized	pubertal induction at 17y	-	4 MI, moderate midface hypoplasia, three hand malformations	breast cancer (37y) (tumour neg. for E2 and P receptor staining and Her-2/neu amplification)
But et al. 2010 (13)	neonatally, diagnosis of ABS (1y), POR diagnosis (7y)	46, XX	p.R457H/ p.R457H	p.R454H/ p.R454H	AI	phallus (1 cm), labial fusion and pigmented with rugae,urogenital sinus, impalpable gonads	prepubertal at 10y	-	5 M, severe midface hypoplasia, moderate craniosynostosis, elbow extension deficit, one hand malformation	maternal virilization in 2nd trimester, consanguineous parents
Idkowiak et al. 2010 (14)	neonatal presentation, diagnosis of partial Androgen insensitivity syndrome, diagnosis at 9y	46, XY	p.E601fsX12/p.Y607C;	p.E598Sfs*12/ p.Y64C	AI	clitoromegaly, urogenital sinus, blind ending vaginal pouch, palpable gonads in inguinal canal => female gender; clitoris growth at 9y	PH1, B1, A1 (9y), clitoral length 3 cm	-	-	delayed bone age (3y behind), growth linear on 50th percentile, parental doubts about gender identification
Idkowiak et al. 2011 (15) & Arlt et al. 2004 (16) (P3)	16 y (r)	46, XX	p.A287P/ p.R457H	p.A284P/ p.R454H	AI	virilized genitalia	Tanner stages PH4, B4, M0 (16y)	rupture of large left ovarian cyst	6 M, moderate midface hypoplasia, hand/feet malformations, elbow extension deficit, additional malformation	-
	12y (r)	46, XX	p.A287P/ p.A287P	p.A284P/ p.A284P	no	clitoromegaly and urogenital sinus	B1, PH2, and M0 => B4, PH3 (13.5y)	bilateral ovarian masses (11.5y) = follicular cysts,	5 M, moderate midface hypoplasia, moderate craniosynostosis, two hand	

								bilaterally recurrent cysts	malformations, elbow extension deficit	
	5y (r)	46, XX	p.A287P/ p.A287P	p.A284P/ p.A284	AI	ambiguous genitalia, raised as a boy until 5y, normal internal female genital organs	induced puberty at 17y, moderate breast development, PH3, B3, M0 (23y)	large ovarian cysts (regress only after glucocorticoid)	5 M, moderate midface hypoplasia, hand/feet malformations, additional malformation	normal psychomotor development, younger sister died at 8y after developing signs + symptoms of shock shortly after undergoing an appendectomy
	16y	46, XX	p.A287P/ p.R223X	p.A284P/ p.R230*	AI	clitoral hypertrophy, urogenital sinus (Prader III).	PH3, B3, and M0 (16y)	primary amenorrhea (16y), infantile uterus and bilateral polycystic ovaries, PH3, B3, M0 (16y)	7 M, complicated midface hypoplasia, moderate craniosynostosis, bilateral radiohumeral synostosis	postnatal ventilation due to respiratory distress (stenosis)
	Neonatal (r) -> 12y	46, XY	p.R457H/ p.Y576X	p.R454H/ p.Y573*	AI	No DSD, undescended testes but palpable in inguinal canal, hypoplastic scrotum	poor genital development at 10.5y, phallus 15mm with little erectile tissue, pubertal induction (10.5y), PH1, G1 (12y) -> PH5, G5 (16y), spontaneous progression of puberty without medication (16y)		9 S, complicated midface hypoplasia, severe craniosynostosis, one hand malformation, severe large joint synostosis	-
	12y (r)	46, XY	p.A287P/ p.P277PfsX65	p.A284P/ c.723-2A>T	AI	No DSD, right testicle undescended but palpable in the inguinal canal	lack of pubertal development (12y), progressed spontaneously; PH1, G1 (12y) -> PH3, G2 (13.5y)		6 M, complicated midface hypoplasia, moderate craniosynostosis, radioulnar synostosis	-
Herkert et al. 2011 (17)	Neonatal, 19y (r)	46, XX	p.T142A/ p.Y376LfsX74	p.T139A/ p.Y373Lfs*74	-	-	axillary and pubic hair = sparse. Normal breast development, infantile internal labia => B5, P3	irregular menses, ovarian cyst (r), lack of libido	4 MI, moderate midface hypoplasia, supination deficit, two hand/feet malformations	-
Flück et al. 2011 (18)	18 months (r)	46, XX	p.P399_E401del/p.P399_E401del	p.P396_E398 del/ p.P396_E398 del	AI	genital virilization (Prader 5)	-	-	MI, presence of mild ABS features	consanguineous parents

	4 y (r)	46, XX	p.P399_E401del/p.P399_E401del	p.P396_E398del/ p.P396_E398del	AI	ambiguous genitalia (Prader 3)	-	-	S, severe ABS phenotype	consanguineous parents
Krone et al. 2012 (19)	Neonatal (r)	46, XX	-		no	DSD	-	-	5 M, severe midface hypoplasia, moderate craniosynostosis, one hand malformation, femoral bowing	-
Arlt et al. 2004 (16) (P1)	Neonatal (r)	46, XX	p.C569Y/ p.Y181D	p.C566Y/ p.Y178D	AI	DSD	-	-	4 MI, moderate midface hypoplasia, three hand malformation	-
Arlt et al. 2004 (16) (P3)	Neonatal (r)	46, XY	p.C569Y/ p.Y181D	p.C566Y/ p.Y178D	AI	-	-	-	0	-
S	Neonatal (r)	46, XX	p.A287P/?	p.A284P/?	AI	DSD	-	-	9 S, severe midface hypoplasia, severe craniosynostosis, hand malformations, large joint synostosis (3)	-
	Neonatal (r)	46, XX	p.A287P/IVS6 -2A>T	p.A284P/ c.723-2A>T	AI	-	-	-	14 S, Complicated midface hypoplasia, complicated craniosynostosis, hand malformation, large joint synostosis, femoral bowing,	anteriorly placed anus, single umbilical artery, dysplastic ears
	Neonatal (r)	46, XY	p.A287P/IVS6 -2A>T	p.A284P/ c.723-2A>T	AI	DSD	-	-	11 S, complicated midface hypoplasia, complicated craniosynostosis, large joint synostosis (3), complicated femoral bowing,	dysplastic ears
	Neonatal (r)	46, XY	p.A287P/ p.V472AfsX102	p.A284P/ c.723-2A>T	AI	DSD	-	-	10 S, severe midface hypoplasia, severe craniosynostosis, two hand malformation, large joint synostosis,	two-vessel umbilical cord
	Neonatal (r)	46, XY	p.Q455RfsX544/IVS7+2dupT	p.Q452Rfs*90/ c.723-2A>T	-	DSD	-	-	12 S, complicated midface hypoplasia, complicated craniosynostosis, large joint synostosis, complicated femoral bowing,	Arnold Chiari malformation
	Neonatal (r)	46, XX	p.A287P/ Del ex U1–1	p.A284P/ Del ex U1–1	AI	-	-	-	4 MI, severe midface hypoplasia, large joint synostosis	-
	Neonatal (r)	46, XX	p.A287P/ IVS8+1G>A	p.A284P/ c.821+1G>A ?	AI	DSD	-	-	9 S, severe midface hypoplasia, severe craniosynostosis, hand/feet	-

									malformation, large joint synostosis	
	Neonatal (r)	46, XX	p.A287P/ p.A287P	p.A284P/ p.A284P	AI	DSD	-	-	7 M, severe midface hypoplasia, severe craniosynostosis, one hand/feet malformation, large joint synostosis	-
	Neonatal (r)	46, XY	p.A287P/ p.A287P	p.A284P/ p.A284P	AI	-	-	-	8 M, severe midface hypoplasia, severe craniosynostosis, hand/feet malformations, extension deficit,	pectus excavatum
	Neonatal (r)	46, XY	p.A287P/ p.G188_V191dup	p.A284P/ p.G185_V188 dup	AI	DSD	-	-	7 M, complicated midface hypoplasia, two hand/feet malformations, complicated femoral bowing	absent left kidney, frontal capillary haemangioma
	Neonatal (r)	46, XX	p.A287P/ p.A287P	p.A284P/ p.A284P	AI	DSD	-	-	0	-
	Neonatal (r)	46, XY	p.A287P /p.I444HfsX6	p.A284P/ p.I441Hfs*6	-	DSD	-	-	10 S, complicated midface hypoplasia, complicated craniosynostosis, large joint synostosis, femoral bowing, scoliosis	no live birth
	Neonatal (r)	46, XX	p.Y87X/	p.Y84*/	AI	DSD	-	-	0	-
	**Stillborn**	46, XX	-			DSD	-	-	14 S, severe midface hypoplasia, severe craniosynostosis, hand/feet malformations, large joint synostosis, complicated femoral bowing	-
	Neonatal (r)	46, XX	p.R498P/ p.R498P	p.R495P/ p.R495P	AI	DSD	-	-	7 M, severe midface hypoplasia, hand/feet malformations, large joint synostosis, femoral bowing	-
	Neonatal (r)	46, XY	p.A287P/ p.R616X	p.A284P/ p.R613*	AI	-	-	-	8 M, severe midface hypoplasia, severe craniosynostosis, one hand/feet malformation, large joint synostosis	-
	31 y	46, XX	p.A287P/ Dup ex 2–5	p.A284P/ Dup ex 2–5	AI	DSD	-	-	6 M, severe midface hypoplasia, hand/feet malformations, large joint synostosis	-

Puiu et al. 2012 (20)	10 months (r)	46, XX	p.A287P /p.A287P	p.A284P /p.A284P	AI?	clitoromegaly, labial fusion, single urogenital orifice (Prader III-IV)	-	-	8 M, severe midface hypoplasia, moderate craniosynostosis, large joint synostosis, hand malformations	maternal virilization, small for gestational age and premature birth; ostium secundum atrial septal defect; mild psychomotor delay and speech difficulties
Guaragna-Filho et al. 2012 (21)	20 months (r)	46, XX	p.R223*/ p.M408K	p.R220*/ p.M405K	AI	complete fusion of labioscrotal folds, virilized genitalia (Prader V), 2.3-cm phallus, without palpable gonads, balanic urethral opening; ovarian cyst (l)	-	-	5 M, craniosynostosis, hand malformations, extension deficit	maternal virilization: voice deepening, clitoromegaly, hirsutism
Sanchez-Garvin et al. 2013 (22)	Neonatal (r)	46, XY	p.G539R/ p.G80R	p.G536R/ p.G77R	AI, high level of K+ and plasma renin activity	ambiguous genitalia, scrotal hypospadias, micropenis, labioscrotal folds, single urethral meatus at median raphe, palpable gonads at inguinal canal	-	-	5 M, severe craniosynostosis, severe midface hypoplasia, additional malformation	dysplastic ears
Oldani et al. 2015 (23)	Prenatally (r)	female	p.A287P/c.732 -2A>T in intron 7	p.A284P/ c.723-2A>T	-	hypoplastic labia majora, vaginal hypoplasia, clitoromegaly, bicornuate uterus	-	-	7 M, moderate craniosynostosis, severe midface hypoplasia, large joint synostosis, femoral bowing	previous pregnancy was terminated because of inferior limb anomalies; this one was terminated as well
Bonamichi et al. 2016 (24)	13 y (r)	46, XX	p.A287P/ p.A287P	p.A284P /p.A284P	Late onset AI	clitoromegaly 2 cm, posterior fusion of major labia (Prader 2)	pubarche 11 y, thelarche at 11.5 y, primary amenorrhea at 13y, infantile uterus and bilateral polycystic ovaries. Induced menarche at 14.5y	-	11 S, severe craniosynostosis, severe midface hypoplasia, large joint synostosis, hand/feet malformation, additional malformations	mild right pyelectasia in sonography, maternal virilization in first (fetal death in 8th month) and this pregnancy; delayed bone age = 10 y, scoliosis, exostosis in clavicles, low bone mineral density => total femur 0.634 g/cm^2 (Z -2.8), femoral neck of 0.617 g/cm^2 (Z

										-2.1) and L1-L4 of 0.676 g/cm^2 (Z -2.4)
Parween et al. 2016 (25)	Neonatal (r)	46, XX	p.L374H/c.-5+4A>G	p.L371H/c.-5+4A>G	AI	posterior labial fusion, rugged major labia, clitoromegaly, no palpable gonads	-	-	0	Prior to birth of patient two spontaneous abortions
Tzetis et al. 2016 (26)	termination at 23 weeks of gestation	female	p.A287P/p.A287P; father: same 7 kb microdeletion in 7q11.23 genomic region (chr7:75608488-75615534 Mb, hg19), deleting exons 4-14	p.A284P /p.A284P	-	clitoromegaly	-	-	6 M, midface hypoplasia, radio-humeral synostosis, hand/feet malformations, additional malformations	found in mother c.831-35C>T, no adrenal hyperplasia
	termination at 17 weeks of gestation	?	p.A287P/p.A287P; p.A287P/NC_000007.13:g.(?_75608488)_(75615534_?)del]	p.A284P /p.A284P	-	-	-	-	0	found in mother c.831-35C>T, no adrenal hyperplasiaC
Koika et al. 2016 (27)	36y (r)	46, XY	p.V531del/p.A259G, poly-morphisms: A503V, C/T; S572S, G/A	p.V528del/p.A259G	Mildly insufficient, mild hypertension	penis (4-5 cm), testicular volume 2-3 ml, hypospadias, hypertrophic foreskin and scrotum bifida	-	azoospermia, infertility	0, mild spinal osteopenia	-
Sengoku et al. 2016 (28)	neonatal	female	p.R457H/p.R457H	p.R454H/p.R454H	-	clitoromegaly	-	-	6 M, craniosynostosis, large joint synostosis, hand/feet malformations	maternal virilization in 2nd trimester, worse at 38. Weeks of gestation, VUR, cephalic angioma => rhinopharyngeal stenosis
Woo et al. 2016 (29)	50 days (r)	male	p.R457H/p.Q555Pfs*19	p.R454H/p.Q552Pfs*19	AI, hyponatriemia, hyperkalemia	bilateral cryptorchidism	-	-	11 S, complicated midface hypoplasia, severe craniosynostosis, hand/feet malformations, bilateral humero-radio-ulnar synostosis, multiple joint synostosis tended to progress with age	deformed ears, progressive respiratory difficulty (14d) => glossoptosis, neuropsychological development is appropriate

Khadilkar et al. 2017 (30)	17y	46, XX	p.G144S/p.W422X	p.G141S/p.W419*	AI	clitoromegaly, (clitoral width of 2.5 cm) with single perineal opening, posterior fourchette fusion, no palpable gonads	B2, P4. genitals = Prader III	primary amenorrhea, bilateral multicystic ovaries	0	
Bai et al. 2017 (31)	27y (r)	46, XX	p.R457H/p.R457H	p.R454H/p.R454H	-	labial fusion	recurrent large ovarian cyst	amenorrhea, bilateral macroovarian cysts	1 MI, one hand malformation	-
Oh et al. 2017 (32)	21 y (r)	female	p.R457H/p.G177R	p.R454H/p.G174R	AI, aldosterone increased in ACTH test	-	secondary amenorrhea, delayed puberty, underdeveloped uterus, loss of pubic hair	-	4 MI, moderate midface hypoplasia, hand/feet malformations, extension deficit	-
Yildiz et al. 2017 (33)	Neonatal (r)	46, XX	(IVS3-1 G>A (c.238-1 G>A)/c.929_937delTCTCGGACT)	c.229-1G>A/ p.I307_S310 delinsT	AI	ambiguous genitalia = genital virilisation (Prader III), 1 cm phallus and impalpable gonads; bilateral ovarian cysts	-	-	6 M, complicated midface hypoplasia, large joint synostosis	maternal virilization, small for gestational age
Song et al. 2018 (34)	28 y	female	p.Y326D/ p.Y326D	p.Y323D/ p.Y33D	-	-	irregular menses (menstrual periods lasting 8–10 days, 30–60 day cycles) or amenorrhea, ovarian cysts	unicornuate uterus and bilateral fallopian tube obstruction	0	IVF, COS, FET
Hao et al. 2018 (35)	1 y	46, XY	p.R223*/ p.R457H	p.R220*/p.R454H	-	micropenis (1.5cm), right cryptorchidism, testicular volume (1 ml)	-	-	10 S, complicated midface hypoplasia, moderate craniosynostosis, one feet malformation, bilateral radiohumeral and -ulnar synostosis, , chest wall deformity, scoliosis, hypotonic bilateral lower extremities	hypertelorism, maternal virilization, mother has Hashimoto's thyroiditis, oligohydramnios
Ono et al. 2018 (36)	Prenatal (r)	46, XY	p.Q201X/ p.R457H	p.Q198*/ p.R454H	-	underdeveloped external genitalia with bilateral testes within a bifid scrotum, penile hypospadias,	-	-	8 M, severe midface hypoplasia, severe craniosynostosis, one hand malformation, bilateral radio-humeral synostosis => dysplastic ears	maternal virilization in 1st and 2nd trimester

						microphallus (2.4 cm)				
Fan et al. 2019 (37)	4.2 y (r)	46, XX	p.R457H/ p.Y248X	p.R454H/ p.Y245*	AI	dysplastic ovaries, infant uterus, phallus (1.5 cm), normal male urethral meatus, fused labioscrotal folds with prominent middle ridgeline	-	-	4 MI, moderate midface hypoplasia, hand malformations, extension deficit	hearing impairment (6 months), maternal virilization
	2.2 y (r)	46, XY	p.R457H/ p.Y248X	p.R454H/ p.Y245*	AI	micropenis (2.6 cm), hypospadias, normal testes volume (2ml)	-	-	3 MI, severe midface hypoplasia, one hand malformation	maternal virilization
	1y (r)	46, XY	p.R457H/ p.R554X	p.R44H/ p.R551*	AI	micropenis (2.3cm), unilateral cryptorchidism (1ml)	-	-	3 MI, moderate midface hypoplasia, one hand malformation, short neck, scoliosis	dysplastic ears, maternal virilization
	6 months (r)	46, XY	p.R457L/ p.Y60	p.R454L/ p.Y604C (LP)	AI	micropenis (1.5cm), coronal hypospadias and testes (1 ml) in hypertrophic scrotum, prominent middle ridgeline	-	-	1 MI, moderate midface hypoplasia	father had micropenis
	3.5 y (r)	46, XY	p.R457H/ p.D210G	p.R454H/ p.D207G	AI	phallus (2.7 cm) with chordee, proximal penile hypospadias, testes bilaterally (1–2 ml) , prominent middle ridgeline			3 MI, moderate midface hypoplasia, hand/feet malformations	slightly delayed bone age
	17.8 y (r)	46, XX	p.R457H/c.517 -19_517- 10delGGCCC CTGTGinsC	p.R454H/ c.51719_517 10delGGCC CCTGTGinsC	AI	labial fusion	B4, P2, ovarian cysts, infantile uterus, amenorrhoea	-	5 M, moderate midface hypoplasia, hand/feet malformations, extension deficit	maternal virilization, bilateral facial paralysis with mild cerebellar atrophy, recurrent otitis media; mild hearing impairment, delayed bone age
	9.8 y (r)	46, XY	p.R457H/ c.517-19_517- 10delGGCCC CTGTGinsC	p.R454H/ c.51719_517 10delGGCC CCTGTGinsC	AI	micropenis (2.5cm), cryptorchidism, unpalpable testes	-	-	5 M, moderate midface hypoplasia, extension deficit, hand/feet malformations	maternal virilization; delayed bone age
	12.5 y (r)	46, XX	p.R457H/p.R4 57H	p.R457H/p.R 457H	AI	clitoromegaly, no vaginal opening, labioscrotal fusion, impalpable gonads	B1, P1, dysplastic ovaries, infantile uterus	-	2 MI, moderate midface hypoplasia, one foot malformation	asymmetric scapula, scoliosis, delayed bone age

Lee et al. 2020 (38)	7 months (r)	46, XX	R457H/ p.I444Hfs*6	R454H/ p.I444Hfs*6	AI	clitoromegaly with partial labial fusion, urogenital sinus	-	-	7 M, severe midfacial hypoplasia, moderate craniosynostosis, bilateral radiohumeral synostosis	stapes otosclerosis due to otitis media, dysplastic ears , conductive hearing loss (both at 7y), vesicoureteral reflux left Grade II (7 months)
	3 months (r)	46, XX	p.R457H/ p.R457H	p.R454H/ p.R454H	AI, high blood pressure at 3y	partially virilized external genitalia with clitoromegaly, partial fusion of the labia majora, urogenital sinus	-	-	0	regression of hypertension after treatment with hydrocortisone
	17 months (r)	46, XY	p.R457H/p.R457H	p.R454H/p.R454H	AI	hypospadias, cryptorchidism, urethral fistula	-	-	0	
	6 months (r)	46, XY	p.R457H/p.I444Hfs*6	p.R454H/p.I444Hfs*6	AI, hypertension 115/69 mmHg [age 28 months]	underdeveloped male genitalia with hypospadias and micropenis	-	-	5 M, midface hypoplasia, moderate craniosynostosis, radiohumeral synostosis, congenital scoliosis	sensory neural hearing loss (both), delayed neurocognitive development (language); VSD, ASD
Zhang et al. 2020 (39)	35 y (r)	female	p.399_401del PSE/IVS14-1G>G/C	p.P396_E398 del/ c.1807-1G>C	Chronic hypertension in pregnancy 12th week	hypoplastic labia majora, vaginal stenosis	B4, sparse axillary, and pubic hair	primary infertility, menstrual irregularity; ovarian cysts/multiple follicular developments, ovulatory dysfunction.	0	twin pregnancy through IVF and FET, preterm, C-section
Song et al. 2020 (40)	10.5 y (r)	46, XX	p.R457H/p.R457H	p.R454H/p.R454H	Hyperkalemia, hyponatremia	clitoromegaly, ovarian cyst at 23 days after birth, vulvular chromatosis	Tanner stage I		6 M, hand/feet malformations, large joint synostosis, scoliosis, low bone mineral density of both feet	hepatosplenomegaly, hereditary spherocytosis, jaundice
Parween et al. 2020 (41)	8 y (r)	46, XX	p.L25Ffs*93/p.R550W	p.L22Ffs*93/ p.R550W	-	rugated labia majora, impalpable gonads, genital tubercle of 1.5 cm with proximal urethral opening = Prader III	-	-	0, delayed bone age (CA = 8y, BA 7y)	maternal virilization in 2nd trimester
Papadakis et al. 2020 (42)	30 y (r)	female	c.1249-1G>C/p.(Pro442Ser) | *incidental*	c.1240-1G>C/ p.P439S	AI	-	spontaneous menarche 13y, oligomenorrhea	primary infertility, irregular menses, multiple macrocysts (10)	0	twin pregnancy

			heterozygous duplication of CYP21A2, associated with a heterozygous non-sense variant (c.952C>T, p.Gln318∗)							
	36 y (r)	female	p.Gln609∗/ p.Trp620Ser	p.Q606*/ p.W617S	AI	-	puberty induced at 16 y, primary amenorrhea, history of macrocysts	primary infertility, oligomenorrhea; suspicion of premature ovarian insufficiency (low AMH, E2)	0	singleton pregnancy, hypertension, preeclampsia, C-section
	33 y (r)	female	p.Gln609∗/ p.Trp620Ser	p.Q606*/ p.W617S	AI	mild genital virilization, labial fusion	spontaneous menarche 16y, history of bilateral macrocysts	primary infertility, oligomenorrhea, dyspareunia	0	
	38 y (r)	female	p.Arg550Trp/p. Leu612_Leu619del	p.R547W/p.L609_L616del	AI	-	spontaneous menarche 12y, oligomenorrhea, macrocysts (3)	primary infertility, secondary amenorrhea after Pill stop, decreased libido, vaginal dryness, bilateral macrocysts	0	
	19 y (r)	female	p.A287P /p.A287P	p.A284P /p.A284P	AI	-	spontaneous menarche 15y, irregular menses (45-50d interval), macrocysts (4)	secondary amenorrhea	3 MI, craniosynostosis, severe midface hypoplasia, arthrogryposis, convergent	strabismus, Marfan-like phenotype
Unal et al. 2021 (43)	8.5 y	46, XX	c.929_937delTCTCGGACT(p.Ile310_Ser313delinsThr)/ c.929_937delTCTCGGACT(p.Ile310_Ser313delinsThr)	p.I307_S310delinsT/ p.I307_S310delinsT	AI	ambiguous genitalia	-	-	ND, ABS phenotype	-
	15 months (r)	46, XY	c.929_937delTCTCGGACT(p.Ile310_Ser313delinsThr)/ c.929_937delT	p.I307_S310delinsT/ p.I307_S310delinsT	AI	-	-	-	1 MI, one hand malformation	-

			CTCGGACT(p.Ile310_Ser313delinsThr)							
Li et al. 2021 (44)	Neonatal (r)	46, XX	p.R457H/exon 4-5 deletion	p.R454H/exon 4-5 deletion	AI	labia majora hypoplasia, labia minora fusion, clitoromegaly, vaginal atresia	-	-	6 M, moderate midface hypoplasia, moderate craniosynostosis, radio-ulnar synostosis, complicated femoral bowing	bronchopneumonia; died 2 months later due to severe pneumonia, dyspnea, congenital heart disease; 2 previous stillborn pregnancies of probands mother
Pan et al. 2021 (45)	29 y	46, XX	p.R457H/p.P339_E401del	p.R547/p.P396_E398del	-	no	normal breast development, no pubic or axillary hair, primary amenorrhea, ovarian cyst (1), infantile uterus (16y)	primary infertility (26-29y); 2014: B4, P2, hypoplastic uterus, thin endometrium, ovarian cyst (right)	1 MI, one hand malformation	twin pregnancy: 1 girl, 1 boy by C-section
Onuki et al. 2021 (46)	7 y	46, XX	p. R457H/ c.760 + 1 G>A	p. R454H/ c.760 + 1 G>A	-	clitoromegaly, labial fusion	-	-	6 M, severe midface hypoplasia, one hand malformation, radiohumeral synostosis, scoliosis (6 months)	-
	7 y	46, XY	p. R457H/ c.396-1 G>A	p. R454H/ c.396-1 G>A	-	left cryptorchidism	-	-	7 M, severe midface hypoplasia, one hand malformation, radiohumeral synostosis, additional, scoliosis (1y)	bilateral middle ear malformations
Li et al. 2021 (47)	10 y (r)	female	p.A541V/ p.Q602*	p.A538V/ p.Q599*	-	clitoromegaly, urogenital sinus	-	-	0, delayed bone age (1 y behind)	initial diagnosis of atypical 21-OHD
Tenenbaum-Rakover et al.	11 y (r)	46, XX	p.G539R/ p.G539R	p.G536R/ p.G536R	AI	fusion of labia majora, small vaginal-urethral orifice	no sign of puberty = P1B1	-	0	consanguineous parents

2021 (48)										
Zhang et al. 2022 (49)	4 y	46, XY	p.G88S /p.G537S	p.G85S /p.G535S	-	short penis, chordee	-	-	4 MI, moderate midface hypoplasia, bilateral radiohumeral synostosis	patient's sibling died 9 hours after birth and had signs of clitoromegaly, maternal virilization at 16 week of gestation
Sato et al. 2022 (50) (double)	47 y	46, XY	p.R457H/ p.L612_W620 delinsR	p.R454H/p.L 609_W617de linsR	hypertensi on	micropenis, left testicular volume 25 ml in the scrotum, right testis located in the inguinal canal => unilateral cryptorchidism	pubic hair = Tanner III, spontaneous puberty	(from 36-47y): semen volumes and sperm concentrations above the lower fifth percentile, possibly infertile	4 MI, one hand malformation, large joint synostosis	bilateral hearing loss, 41y HbA1c 6.5%, Previously reported as patient by Fukami et al. 2005 (5)
Li et al. 2022 (51)	35 y (r)	female	p.R457H/ p.L454P	p.R454H/p.L 451P	Hypertensi on at 20 weeks of gestation	no	menarche at 14y, oligomenorrhea until hormone replacement therapy; B4, P2	primary infertility, bilateral ovarian cysts, with IVF and FET = a healthy baby at 36 weeks per c-section, endometritis	0	patient's sister has frequent ovarian cysts and received operations twice
	36 y (r)	female	p.I544T /p.A575T	p.I544T/ p.A572T		narrow vagina, incomplete transverse vaginal septum	primary amenorrhea, B5, bilateral ovarian cysts	primary infertility, had already 2 FET, early pregnancy loss, endometritis, miscarried in seventh week	1 MI, one hand malformation	elevated levels of thyroid peroxidase auto-antibody (Anti-TPOAb), Antib2-GPI antibody, and anti-cardiolipin antibody (ACA)
Gusman o et al. 2022 (52)	Neonatal (r)	male	p.V631I/c.516 G>A	p.V628I/ p.A190=	AI	-	TART at 14y	oligoasthenoterat ozoospermia (at 20y), hypoposia, bilateral TART (18y -> 20y grown in size), spontaneous paternity at 22y	0	-
Charoen sri et al. 2023 (53)	35 y (r)	female	p.G539R/c.166 9+5_1669+32 del	p.G536R/ c.1660+5_16 60+32del	-	-	late menarche	irregular cycles, unruptured ovarian follicles/cysts, infertility	0	-
Baranow ski et al. 2025 (54)	Neonatal (r)	female	p.E217del/ p.E217del	p.E214del/ p.E214del	-	ambiguous genitalia; posterior fusion of the labia minora and	-	-	13 S, complicated midface hypoplasia, moderate craniosynostosis, hand/feet malformations, radio-	bilateral atretic ear canals, saw ABS in ultrasound at 18.5 weeks gestation.

						a single urethral opening			humeral synostosis, large joint synostosis, complicated femoral bowing	consanguineous parents, Birth weight = 2.5kg, patient died 12 hours after birth as oxygen levels desaturated
Zhang et al. 2025 (55)	12 y (r)	46, XX	p.R457H/ p.R457H	p.R454H/ p.R454H	-	no	recurrent ovarian cysts; B2, P3/2 (pubic/axillary)	-	1 MI, scoliosis	
	14 y (r)	46, XX	p.R457H/ p.R457H	p.R454H/ p.R454H	AI	Prader III	pelvic mass, irregular menses (2-6 months); B2, P4/3	-	2 MI, midface hypoplasia, one foot malformation	
	19 y (r)	46, XX	p.R457H/ p.R457H	p.R454H/ p.R454H	-	Prader IV	primary amenorrhea; B3, P3/3		2 MI, radiohumeral synostosis	
	17 y (r)	46, XX	p.R457H/ p.R457H	p.R454H/ p.R454H	-	No	primary amenorrhea; B5, P2/2	-	3 MI, large joint synostosis (3)	
	17 y (r)	46, XX	p.R457H/ p.R457H	p.R454H/ p.R454H	-	Prader I	irregular menses (2-3 months); B3, P4/2	-	2 MI, one hand malformation, scoliosis	
	16 y (r)	46, XX	p.R457H/ p.R457H	p.R454H/ p.R454H	-	Prader III	secondary amenorrhea, menses (1-2 months); B4, P2/2	-	1 MI, midface hypoplasia	
	11 y (r)	46, XX	p.R457H/ p.Gu562Gly fs*13	p.R454H/ p.E559Gfs*13	AI	no	recurrent ovarian cysts; B2, P1/2	-	1 MI, midface hypoplasia	
	31 y (r)	46, XX	Exon5-exon6 Del/p.*681Leu fs*24	Exon5-exon6 Del/ p.*678Lext*23	-	no	ovarian cysts; B5, P3/2	primary amenorrhea	0	torticollis
	12 y (r)	46, XX	p.R457H/ p.R457H	p.R454H/ p.R454H	-	no	ovarian cysts, menses (2-4 months); B3, P1/2	-	0	maternal virilization
	24 y (r)	46, XX	p.R457H/ p.R457H	p.R454H/ p.R454H	-	no	ovarian cysts; B5, P1/2	secondary amenorrhea (2-6 months)	0	
	7 y (r)	46, XX	p.R457H/ p.R457H	p.R454H/ p.R454H	-	Prader IV	-	-	2 MI, midface hypoplasia, one hand malformation	
	17 y (r)	46, XX	p.R457H/ p.R457H	p.R454H/ p.R454H	-	Prader III	ovarian cysts, secondary amenorrhea (1-3 months); B3-4, P2/2	-	2 MI, midface hypoplasia, one hand malformation	

AI, adrenal insufficiency; ABS, Antley-Bixler syndrome; M,moderate; MI, mild; ND, not defined, S, severe; EEG, electroencephalography; BA, bone age; CA, chronological age; VUR, Vesicoureteral reflux; GERD, Gastroesophageal reflux disease; NICU, neonatal intensive care unit; E2, oestrogen; P, progesterone; 17-OHP, 17α-Hydroxyprogesterone; GnRH, Gonadotropin-Releasing Hormon; UTI, Urinary tract infection; i.m., intramuscular; p.o., per os; hCG, Human chorionic gonadotropin; hMG, Human menopausal gonadotropin; IVF, In-vitro-fertilisation; FET, Frozen embryo transfer; HGVS; Human Genome Variation Society, HRT, Hormonal replacement therapy; COC, combined oral contraceptive; 21-OHD, 21-hydroxylase deficiency; VSD, ventricular septal defect; ASD, atrial septal defect

Table S 2: Phenotypes of reported polymorphisms in POR-partner genes

	POR-Partner	Phenotype	Evidence - Study Type	Ref.
Cancer	*CYP1A1*	Acute lymphoblastic leukemia	Meta-analysis	(56)
			Clinical case-control genetic association	(57)
			Clinical meta-analysis	(58)
		Biliary tract cancer	Clinical case-control genetic association (Chinese population)	(59)
		Endometrial cancer		(60)
			Clinical case-control genetic association)	(61)
		Esophageal cancer	Clinical case-control genetic association (Chinese population)	(62)
			Clinical case-control genetic association (tobacco smoking)	(63)
			Meta-analysis	(64)
		Oral cancer	Clinical case-control genetic association (Asian population)	(65) (66)
		Head and neck cancer	Clinical case-control genetic association	(67)
		Lung cancer	Clinical case-control genetic association (tobacco smoking)	(68)
			Clinical case-control genetic association (tobacco smoking, Egyptian population)	(69)
			Meta-analysis	(70)
		Prostate cancer	Clinical case-control genetic association	(71)
			Meta-analysis	(72)
		Renal cancer	Clinical case-control genetic association (increase susceptibility in smokers, Chinese populations)	(73)
		Stomach cancer	Clinical case-control genetic association	(74)
		Myeloid leukemia	Clinical case-control genetic association (Sudanese population)	(75)
		Laryngeal cancer	Clinical case-control genetic association (Asian population)	(76)
			Meta-analysis (Asian population)	(62)
		Cervical cancer	Clinical case-control genetic association (women)	(77)
		Bladder cancer	Meta-analysis	(78)
		Colorectal cancer	Meta-analysis	(79)

Cancer		Breast cancer	Meta-analysis	(80)
			Clinical case-control genetic association (Korean women)	(81)
		Adult leukemia	Meta-analysis	(82)
		Gastric cancer	Clinical case-control genetic association	(83)
	CYP1A2	Breast cancer	Clinical case-control genetic association	(84)
		Bladder cancer	Clinical case-control genetic association	(85)
	CYP1B1	Lung cancer	Clinical case-control genetic association	(86)
		Breast cancer	Clinical case-control genetic association	(86) (87)
		Prostate cancer	Clinical case-control genetic association	(88)
			Meta-analysis	(89)
		Urinary cancer	Meta-analysis	(90)
		Laryngeal cancer	Clinical case-control genetic association	(91)
	CYP2A13	Lung cancer	Clinical case-control genetic association	(92)
	CYP2B6	Acute myeloid leukaemia	Clinical case-control genetic association	(93)
		Head and neck cancer	Genome wide association study (GWAS)	(94)
		Acute leukemia	Clinical case-control genetic association (Chinese population)	(95)
	CYP2C8	Bladder cancer	Clinical case-control genetic association	(96)
	CYP2C9	Head and neck cancer	Clinical case-control genetic association	(97)
		Gastric cancer	Cross-sectional observational study	(98)
	CYP2C19	Breast cancer	Clinical pharmacogenetic study (genetic association & drug metabolism)	(99)
		Hepatocellular carcinoma	Clinical case-control genetic association	(100)
		Head and neck cancer	Clinical case-control genetic association	(97)
		Multiple myeloma	Clinical pharmacogenetic study (genetic association & drug metabolism)	(100)
	CYP2D6	Acute lymphoblastic leukemia	Clinical case-control genetic association	(101)
			Clinical case-control genetic association (Indian population)	(102)
		Acute myeloid leukaemia	Clinical pharmacogenetic study (genetic association & drug metabolism)	(103)
		Head and neck cancer	Clinical case-control genetic association	(97)
		Prostate cancer	Clinical case-control genetic association (Indian population)	(104)
		Breast cancer	Clinical case genetic association (Thai population)	(105)
	CYP2E1	Collateral cancer	Clinical case-control genetic association (red meat intake)	(106)

**Cancer**		Hepatocellular carcinoma	Clinical case-control genetic association (alcohol consumption)	(107)
			Clinical case-control genetic association	(108)
		Urinary cancer	Meta-analysis	(109)
		Nasopharyngeal cancer	Clinical case-control genetic association	(110)
		Sporadic colorectal cancer	Clinical case-control genetic association	(111)
		Colorectal cancer	Clinical case-control genetic association (Kashmiri population)	(112)
		Gastrointestinal cancer	Clinical case-control genetic association	(113)
		Esophageal cancer	Meta-analysis	(114)
		Acoustic neuroma	Clinical case-control genetic association	(115)
		Cervical cancer	Clinical case-control genetic association	(113)
	CYP2W1	Colorectal cancer	Clinical case-control genetic association	(116)
	CYP3A4	Lung cancer	Meta-analysis	(117)
		Prostate cancer	Meta-analysis and meta-regression	(118)
		Acute myeloid leukaemia	Clinical case-control genetic association	(119)
	CYP3A5	Chronic myeloid leukaemia	Clinical case-control genetic association (Indian population)	(120)
		Prostate cancer	Meta-analysis	(121)
		Acute myeloid leukaemia	Clinical case-control genetic association	(122)
	CYP3A5	Chronic myeloid leukaemia	Clinical case-control genetic association (Indian population)	(120)
		Prostate cancer	Meta-analysis	(121)
		Acute myeloid leukaemia	Clinical case-control genetic association	(122)
	CYP3A43	Prostate cancer	Clinical case-control genetic association	(123)
	CYP4B1	Breast cancer	Clinical case-control genetic association (Chinese women)	(124)
		Gastric cancer	Clinical case-control genetic association (Chinese population)	(125)
		Lung cancer	Clinical case-control genetic association (Chinese women)	(126)
	CYP4F2	Lung cancer	Clinical case-control genetic association (Chinese women)	(127)
	CYP4F12	Glioma	Genome wide association study (GWAS) (Chinese population)	(128)
		Bladder cancer	Meta-analysis	(129)
	CYP17A1		Clinical case-control genetic association	(130)
		Breast cancer		
		Non-Hodgkin lymphoma		
		Hepatocellular carcinoma		
		Prostate cancer	Clinical case-control genetic association (Korean men)	(131)

	CYP19A1	Breast cancer	Clinical case-control genetic association (Chinese women)	(132)
			Clinical case-control genetic association	(133)
		Endometrial cancer	Clinical case-control genetic association (Russian women)	(134)
		Esophageal adenocarcinoma	Clinical case-control genetic association	(135)
		Ovarian cancer	Clinical case-control genetic association (Russian women)	(134)
		Prostate cancer	Meta-analysis	(136)
		Lung cancer	Clinical case-control genetic association	(137)
		Bladder cancer	Clinical case-control genetic association (Chinese population)	(138)
	CYP26B1	Colorectal cancer	Clinical case-control genetic association	(139)
		Esophageal cancer (?)	Exome wide case-control genetic association	(140)
	CYB5A	Breast cancer	Clinical case-control genetic association (African American population)	(141)
	**HMOX1**	Lung adenocarcinoma	Clinical case-control genetic association	(142)

Cardiovascular system	***CYP1A1***	(Essential) hypertension	Clinical case-control genetic association	(143)
		Coronary artery disease	Clinical case-control genetic association	(144)
	CYP2C9	Hypertension	Clinical pharmacogenetic study (genetic association & drug metabolism)	(145)
	CYP2J2	Essential hypertension	Clinical case-control genetic association	(146)
		Coronary artery disease	Meta-analysis	(147)
		Ischemic stroke	Clinical case-control genetic association (Chinese population)	(148)
		Myocardial infraction	Clinical study (case-control genetic association, tobacco smoking)	(149)
	CYP2R1	Hypertension	Clinical genetic study (Mendelian randomisation)	(150)
			Clinical case-control genetic association (Chinese population)	(151)
		Coronary artery disease	Clinical case-control genetic association (Chinese population)	(152)
		Myocardial infraction	Clinical case-control genetic association (Egyptian population)	(153)
		Ischemic stroke	Clinical genetic study (UK Biobank population)	(154)
	CYP3A4	Hypertension	Clinical case-control genetic association (Chinese population)	(155)
		Coronary artery disease	Clinical case-control genetic association (Chinese population)	(156)
	CYP3A5	Essential hypertension	OMIM:145500	(157)
		Myocardial infraction	Clinical case-control genetic association	(158)

Cardiovascular system	***CYP4A11***	(Essential) hypertension	Clinical case-control genetic association	(159, 160)
			Clinical case-control genetic association (Chinese population)	(160, 161)
		Coronary artery disease	Clinical case-control genetic association	(162)
			Clinical case-control genetic association (Chinese population)	(163)
			Clinical case-control genetic association (Japanese population)	(164)
	CYP2C19	Essential hypertension	Clinical case-control genetic association	(165)
		Myocardial infraction	Clinical case-control genetic association	(158)
		Ischemic stroke	Clinical case-control genetic association	(166)
	CYP4A22	Coronary artery disease	Clinical case-control genetic association	(167)
	CYP4F2	Ischemic stroke	Clinical case-control genetic association	(168)
			Meta-analysis	(169)
		Coronary artery disease	Clinical case-control genetic association (Chinese population)	(170)
		Myocardial infraction	Clinical case-control genetic association (in men)	(171)
	CYP4V2	Ischemic stroke	Clinical case-control genetic association (Chinese population)	(172)
		Coronary artery disease	Clinical case-control genetic association (Chinese population)	(173)
	CYP7A1	Hypertension	Clinical case-control genetic association (Chinese population)	(174)
		Coronary artery disease	Clinical case-control genetic association	(175)
		Acute coronary syndrome	Clinical case-control genetic association (Mexican population)	(176)
	CYP17A1	Essential hypertension	Clinical case-control genetic association (Chinese population)	(72)
		Hypertension	Meta-analysis	(177)
		Preeclampsia	Clinical case-control genetic association (Chinese population)	(178)
				(179)
		Coronary artery disease	Clinical case-control genetic association (Chinese population)	(180)
	CYP19A1	Coronary artery disease	Clinical case-control genetic association	(181)
			Clinical case-control genetic association (Chinese population)	(182)
		Preeclampsia	Clinical case-control genetic association (Chinese women)	(178)
		Atherosclerotic cardiovascular disease	Clinical case-control genetic association (Postmenopausal women)	(183)
	CYP7B1	Coronary artery disease	Clinical case-control genetic association (Hainan Han population)	(184)

	HMOX1	Abdominal aortic aneurysm	Clinical case-control genetic association	(185)
		Essential hypertension	Clinical case-control genetic association (Japanese women)	(186)
Endocrine & Metabolic system	***CYP1A1***	Polycystic ovary syndrome (PCOS)	Clinical case-control genetic association	(187)
	CYP1B1	Type 2 diabetes	Clinical case-control genetic association (Saudi Arabia)	(188)
	CYP2E1	Type 2 diabetes	Clinical case-control genetic association (Chinese population)	(189)
	CYP2C8	Type 2 diabetes	Clinical case-control genetic association (Indian population)	(190)
	CYP2C9	Type 2 diabetes	Clinical case-control genetic association (Indian population)	(190)
	CYP2R1	Type 1 diabetes	Clinical case-control genetic association	(191)
		Metabolic syndrome components	Cross-sectional observational study	(192)
	**CYP2C19**	Type 2 diabetes	Clinical case-control genetic association (Chinese population)	(193)
	CYP3A4	Type 2 diabetes	Clinical case-control genetic association (Japanese population)	(194)
	CYP7A1	Hypercholesterolemia	ORPHA:209902	
		Type 2 diabetes	Clinical case-control genetic association (Chinese population)	(137)
	CYP8B1	Insulin sensitivity	Clinical case-control genetic association and functional	(195)
	CYP17A1	Polycystic ovary syndrome (PCOS)	Systematic review and meta-analysis	(196)
			Meta-analysis	(197)
		Type 2 diabetes	Clinical case-control genetic association (Chinese population)	(198)
	CYP19A1	Polycystic ovary syndrome (PCOS)	Clinical case-control genetic association	(199)
		Obesity-related phenotypes	Clinical case-control genetic association (Chinese women)	(200)
	HMOX1	Metabolic syndrome components	Clinical case-control genetic association (Mexican population)	(201)

Respiratory system	***CYP1A1***	Chronic obstructive pulmonary disease	Meta-analysis (Asian population)	(202)
			Clinical case-control genetic association	(203)
			Clinical case-control genetic association	(84)
	**CYP1A2**	Chronic obstructive pulmonary disease	Clinical case-control genetic association	(84)
	CYP2E1	Chronic obstructive pulmonary disease	Clinical case-control genetic association	(204)
	CYP2B6	Chronic obstructive pulmonary disease	Clinical case-control genetic association (Chinese population)	(205)
	CYP2D6	Asthma	Clinical case-control genetic association	(206)
	CYP3A4	Chronic obstructive pulmonary disease	Clinical case-control genetic association (Hainan Han population)	(207)
	CYP4F2	Chronic obstructive pulmonary disease	Clinical case-control genetic association	(208)
	**HMOX1**	Chronic obstructive pulmonary disease	OMIM:606963 (in smokers)	(209)<br>(210)<br>(211)
		Adult respiratory distress syndrome	Clinical case-control genetic association	(212)
Reproductive system	***CYP1A1***	Recurrent pregnancy loss	Meta-analysis (Asian population)	(213)
		Male infertility	Clinica case-control genetic association	(214)
		Endometriosis	Clinica case-control genetic association	(215)
	CYP2C19	Endometriosis	Clinical case-control genetic association	(216)
	CYP19A1	Endometriosis	Clinical case-control genetic association (Chinese population)	(217)
	CYP17A1	Endometriosis (?)	Clinical case-control genetic association	(218)
	HMOX1	Endometriosis	Clinical case-control genetic association (Polish population)	(219)
Nervous	***CYP2R1***	Multiple sclerosis	Clinical case-control genetic association	(220)
	CYP2D6	Parkinson’s disease	Clinical case-control genetic association (Pakistan)	(221)
	**CYP4A22**	Stroke	Clinical case-control genetic association (Chinese population)	(222)
	**CYP17A1**	Alzheimer’s disease	Clinical case-control genetic association	(223)
	**CYP19A1**	Alzheimer’s disease	Clinical case-control genetic association (Chinese population)	(224)

	**CYP46A1**	Alzheimer's disease	Clinical case-control genetic association	(225)
			Clinical case-control genetic association (Chinese population)	(226)
			Meta-analysis (Chinese population)	(227)
Musculoskeletal	***CYP1A1***	Reactive arthritis	Clinical case-control genetic association	(228)
		Psoriatic arthritis	Clinical case-control genetic association	(229)
	CYP17A1	Osteoporosis	Clinical case-control genetic association (Postmenopausal women, Turkey)	(230)
	CYP19A1	Osteoporosis	Clinical case-control genetic association	(231)
			Clinical case-control genetic association (Postmenopausal women, Turkey)	(230)
	CYP1B1	Osteoporosis	Clinical case-control genetic association (Postmenopausal American women)	(232)

References

1. Flück CE, Tajima T, Pandey AV, Arlt W, Okuhara K, Verge CF, et al. Mutant P450 oxidoreductase causes disordered steroidogenesis with and without Antley-Bixler syndrome. Nat Genet. 2004;36(3):228–30.
2. Adachi M, Tachibana K, Asakura Y, Yamamoto T, Hanaki K, Oka A. Compound heterozygous mutations of cytochrome P450 oxidoreductase gene (POR) in two patients with Antley-Bixler syndrome. Am J Med Genet A. 2004;128A(4):333–9.
3. Wudy SA, Hartmann MF, Draper N, Stewart PM, Arlt W. A male twin infant with skull deformity and elevated neonatal 17-hydroxyprogesterone: a prismatic case of P450 oxidoreductase deficiency. Endocr Res. 2004;30(4):957–64.
4. Huang N, Pandey AV, Agrawal V, Reardon W, Lapunzina PD, Mowat D, et al. Diversity and function of mutations in p450 oxidoreductase in patients with Antley-Bixler syndrome and disordered steroidogenesis. Am J Hum Genet. 2005;76(5):729–49.
5. Fukami M, Horikawa R, Nagai T, Tanaka T, Naiki Y, Sato N, et al. Cytochrome P450 oxidoreductase gene mutations and Antley-Bixler syndrome with abnormal genitalia and/or impaired steroidogenesis: molecular and clinical studies in 10 patients. J Clin Endocrinol Metab. 2005;90(1):414–26.
6. Fukami M, Hasegawa T, Horikawa R, Ohashi T, Nishimura G, Homma K, et al. Cytochrome P450 oxidoreductase deficiency in three patients initially regarded as having 21-hydroxylase deficiency and/or aromatase deficiency: diagnostic value of urine steroid hormone analysis. Pediatr Res. 2006;59(2):276–80.
7. Williamson L, Arlt W, Shackleton C, Kelley RI, Braddock SR. Linking Antley-Bixler syndrome and congenital adrenal hyperplasia: a novel case of P450 oxidoreductase deficiency. Am J Med Genet A. 2006;140A(17):1797–803.
8. Hershkovitz E, Parvari R, Wudy SA, Hartmann MF, Gomes LG, Loewental N, et al. Homozygous mutation G539R in the gene for P450 oxidoreductase in a family previously diagnosed as having 17,20-lyase deficiency. J Clin Endocrinol Metab. 2008;93(9):3584–8.
9. Sahakitrungruang T, Huang N, Tee MK, Agrawal V, Russell WE, Crock P, et al. Clinical, genetic, and enzymatic characterization of P450 oxidoreductase deficiency in four patients. J Clin Endocrinol Metab. 2009;94(12):4992–5000.
10. Ko JM, Cheon CK, Kim GH, Yoo HW. A case of Antley-Bixler syndrome caused by compound heterozygous mutations of the cytochrome P450 oxidoreductase gene. Eur J Pediatr. 2009;168(7):877–80.
11. Iijima S. Cytochrome P450 Oxidoreductase Deficiency with Antley-Bixler Syndrome: Steroidogenic Capacities. 2009.
12. Tomalik-Scharte D, Maiter D, Kirchheiner J, Ivison HE, Fuhr U, Arlt W. Impaired hepatic drug and steroid metabolism in congenital adrenal hyperplasia due to P450 oxidoreductase deficiency. Eur J Endocrinol. 2010;163(6):919–24.
13. But WM. Ambiguous genitalia, impaired steroidogenesis, and Antley-Bixler syndrome in a patient with P450 oxidoreductase deficiency. 2010.
14. Idkowiak J, Malunowicz EM, Dhir V, Reisch N, Szarras-Czapnik M, Holmes DM, et al. Concomitant mutations in the P450 oxidoreductase and androgen receptor genes presenting with 46,XY disordered sex development and androgenization at adrenarche. J Clin Endocrinol Metab. 2010;95(7):3418–27.
15. Idkowiak J, O'Riordan S, Reisch N, Malunowicz EM, Collins F, Kerstens MN, et al. Pubertal presentation in seven patients with congenital adrenal hyperplasia due to P450 oxidoreductase deficiency. J Clin Endocrinol Metab. 2011;96(3):E453–62.
16. Arlt W, Walker EA, Draper N, Ivison HE, Ride JP, Hammer F, et al. Congenital adrenal hyperplasia caused by mutant P450 oxidoreductase and human androgen synthesis: analytical study. The Lancet. 2004;363(9427):2128–35.

17. Herkert JC, Blaauwwiekel EE, Hoek A, Veenstra-Knol HE, Kema IP, Arlt W, et al. A rare cause of congenital adrenal hyperplasia: Antley-Bixler syndrome due to POR deficiency. Neth J Med. 2011;69(6):281–3.
18. Fluck CE, Mallet D, Hofer G, Samara-Boustani D, Leger J, Polak M, et al. Deletion of P399_E401 in NADPH cytochrome P450 oxidoreductase results in partial mixed oxidase deficiency. Biochem Biophys Res Commun. 2011;412(4):572–7.
19. Krone N, Reisch N, Idkowiak J, Dhir V, Ivison HE, Hughes BA, et al. Genotype-phenotype analysis in congenital adrenal hyperplasia due to P450 oxidoreductase deficiency. J Clin Endocrinol Metab. 2012;97(2):E257–67.
20. Puiu M. A Case of Antley Bixler Syndrome: Diagnosis and Outcome. Acta Endocrinologica (Bucharest). 2012;8(3):479–84.
21. Guaragna-Filho G. 46,XX DSD and Antley-Bixler syndrome due to novel mutations in the cytochrome P450 oxidoreductase gene. 2012.
22. Sanchez-Garvin D, Albaladejo S, Ezquieta B, Corripio R. Disorder of sex development as a diagnostic clue in the first Spanish known newborn with P450 oxidoreductase deficiency. BMJ Case Rep. 2013;2013.
23. Oldani E, Garel C, Bucourt M, Carbillon L. Prenatal Diagnosis of Antley-Bixler Syndrome and POR Deficiency. Am J Case Rep. 2015;16:882–5.
24. Bonamichi BD, Santiago SL, Bertola DR, Kim CA, Alonso N, Mendonca BB, et al. Long-term follow-up of a female with congenital adrenal hyperplasia due to P450-oxidoreductase deficiency. Arch Endocrinol Metab. 2016;60(5):500–4.
25. Parween S, Roucher-Boulez F, Fluck CE, Lienhardt-Roussie A, Mallet D, Morel Y, et al. P450 Oxidoreductase Deficiency: Loss of Activity Caused by Protein Instability From a Novel L374H Mutation. J Clin Endocrinol Metab. 2016;101(12):4789–98.
26. Tzetis M, Konstantinidou A, Sofocleous C, Kosma K, Mitrakos A, Tzannatos C, et al. Compound heterozygosity of a paternal submicroscopic deletion and a maternal missense mutation in POR gene: Antley-bixler syndrome phenotype in three sibling fetuses. Birth Defects Res A Clin Mol Teratol. 2016;106(7):536–41.
27. Koika V, Armeni AK, Georgopoulos NA. Delayed diagnosis of disorder of sex development (DSD) due to P450 oxidoreductase (POR) deficiency. Hormones (Athens). 2016;15(2):277–82.
28. Sengoku K, Kawamura M, Tanahashi Y, Matsuo K, Furuya A, Takeguch R, et al. P450 oxidoreductase deficiency with maternal virilization during pregnancy. Clinical and Experimental Obstetrics & Gynecology. 2016;43(6):902–4.
29. Woo H, Ko JM, Shin CH, Yang SW. Two cases of Antley-Bixler syndrome caused by mutations in different genes, FGFR2 and POR. Journal of Genetic Medicine. 2016;13(1):31–5.
30. Khadilkar KS, Jagtap V, Lila A, Bandgar T, Shah NS. Cytochrome P450 Oxidoreductase Deficiency: Novel Cause of Ambiguity with Primary Amenorrhea. Indian J Endocrinol Metab. 2017;21(2):360–2.
31. Bai Y, Li J, Wang X. Cytochrome P450 oxidoreductase deficiency caused by R457H mutation in POR gene in Chinese: case report and literature review. J Ovarian Res. 2017;10(1):16.
32. Oh J, Song JS, Park JE, Jang SY, Ki CS, Kim DK. A Case of Antley-Bixler Syndrome With a Novel Likely Pathogenic Variant (c.529G>C) in the POR Gene. Ann Lab Med. 2017;37(6):559–62.
33. Yildiz M. A Case of 46,XX DSD Due to a Novel Mutation in P450 Oxidoreductase Gene. 2017.
34. Song T, Wang B, Chen H, Zhu J, Sun H. In vitro fertilization-frozen embryo transfer in a patient with cytochrome P450 oxidoreductase deficiency: a case report. Gynecol Endocrinol. 2018;34(5):385–8.
35. Hao C, Guo J, Guo R, Qi Z, Li W, Ni X. Compound heterozygous variants in POR gene identified by whole-exome sequencing in a Chinese pedigree with cytochrome P450 oxidoreductase deficiency. Pediatr Investig. 2018;2(2):90–5.
36. Ono H, Numakura C, Homma K, Hasegawa T, Tsutsumi S, Kato F, et al. Longitudinal serum and urine steroid metabolite profiling in a 46,XY infant with prenatally identified POR deficiency. J Steroid Biochem Mol Biol. 2018;178:177–84.

37. Fan L, Ren X, Song Y, Su C, Fu J, Gong C. Novel phenotypes and genotypes in Antley-Bixler syndrome caused by cytochrome P450 oxidoreductase deficiency: based on the first cohort of Chinese children. Orphanet J Rare Dis. 2019;14(1):299.
38. Lee Y, Choi JH, Oh A, Kim GH, Park SH, Moon JE, et al. Clinical, endocrinological, and molecular features of four Korean cases of cytochrome P450 oxidoreductase deficiency. Ann Pediatr Endocrinol Metab. 2020;25(2):97–103.
39. Zhang T, Li Z, Ren X, Huang B, Zhu G, Yang W, et al. Clinical and genetic analysis of cytochrome P450 oxidoreductase (POR) deficiency in a female and the analysis of a novel POR intron mutation causing alternative mRNA splicing : Overall analysis of a female with POR deficiency. J Assist Reprod Genet. 2020;37(10):2503–11.
40. Song F, Feng S, Shen X, Du M, Yin H, Liu R, et al. Next-Generation Sequencing Revealed Disease-Causing Variants in Two Genes in a Patient With Combined Features of Spherocytosis and Antley-Bixler Syndrome With Genital Anomalies and Disordered Steroidogenesis. Front Genet. 2020;11:976.
41. Parween S, Fernandez-Cancio M, Benito-Sanz S, Camats N, Rojas Velazquez MN, Lopez-Siguero JP, et al. Molecular Basis of CYP19A1 Deficiency in a 46,XX Patient With R550W Mutation in POR: Expanding the PORD Phenotype. J Clin Endocrinol Metab. 2020;105(4).
42. Papadakis GE, Dumont A, Bouligand J, Chasseloup F, Raggi A, Catteau-Jonard S, et al. Non-classic cytochrome P450 oxidoreductase deficiency strongly linked with menstrual cycle disorders and female infertility as primary manifestations. Hum Reprod. 2020;35(4):939–49.
43. Unal E, Demiral M, Yildirim R, Tas FF, Ceylaner S, Ozbek MN. Cytochrome P450 oxidoreductase deficiency caused by a novel mutation in the POR gene in two siblings: case report and literature review. Hormones (Athens). 2021;20(2):293–8.
44. Li H, Zhao A, Xie M, Chen L, Wu H, Shen Y, et al. Antley-Bixler syndrome arising from compound heterozygotes in the P450 oxidoreductase gene: a case report. Transl Pediatr. 2021;10(12):3309–18.
45. Pan P, Zheng L, Chen X, Huang J, Yang D, Li Y. Successful live birth in a Chinese woman with P450 oxidoreductase deficiency through frozen-thawed embryo transfer: a case report with review of the literature. J Ovarian Res. 2021;14(1):22.
46. Onuki T, Ohtsu Y, Hiroshima S, Sawano K, Nagasaki K. Two cases of cytochrome P450 oxidoreductase deficiency with severe scoliosis and surgery requirement. Congenit Anom (Kyoto). 2021;61(5):202–3.
47. Li Z, Liang Y, Du C, Yu X, Hou L, Wu W, et al. Clinical applications of genetic analysis and liquid chromatography tandem-mass spectrometry in rare types of congenital adrenal hyperplasia. BMC Endocr Disord. 2021;21(1):237.
48. Tenenbaum-Rakover Y, Admoni O, Elias-Assad G, London S, Noufi-Barhoum M, Ludar H, et al. The evolving role of whole-exome sequencing in the management of disorders of sex development. Endocr Connect. 2021;10(6):620–9.
49. Zhang J, Woo KL, Hai Y, Wang S, Lin Y, Huang Y, et al. Congenital adrenal hyperplasia due to P450 oxidoreductase deficiency. Front Endocrinol (Lausanne). 2022;13:1020880.
50. Sato T, Ishii T, Fukami M, Ogata T, Hasegawa T. The first adult case of cytochrome P450 oxidoreductase deficiency with sufficient semen volume and sperm concentration. Congenit Anom (Kyoto). 2022;62(3):136–7.
51. Li Y, Zhang CL, Zhang SD. Infertility treatment for Chinese women with P450 oxidoreductase deficiency: Prospect on clinical management from IVF to FET. Front Endocrinol (Lausanne). 2022;13:1019696.
52. Gusmano C, Calogero AE, Cannarella R. Heterozygous POR gene mutations in a patient with congenital adrenal hyperplasia. Endocrine. 2022;77(2):401–2.
53. Charoensri S, Thakur M, Auchus RJ. A Successful Ovulation Induction And Embryo Generation In A Woman With P450-oxidoreductase Deficiency From Mutation G539R, Which Preferentially Impairs 17,20-lyase Activity. 2023.

54. Baranowski ES, Idkowiak J, Waterson J, D'Harlingue A, Olney AH, Ivison HE, et al. The distinctive P450 oxidoreductase (PORD) urinary steroid metabolome in the first week of life: Report of three cases with severe disorder. J Steroid Biochem Mol Biol. 2025;251:106760.
55. Zhang D, Ding L, Deng S, Tian Q. Clinical Characteristics and Molecular Aetiology of Cytochrome P450 Oxidoreductase Deficiency Diagnosed in 46,XX Patients. Reprod Sci. 2025;32(7):2474–83.
56. Frikha I, Frikha R, Medhaffer M, Charfi H, Turki F, Elloumi M. Impact of CYP1A1 variants on the risk of acute lymphoblastic leukemia: evidence from an updated meta-analysis. Blood Res. 2024;59(1):9.
57. Agha A, Shabaan H, Abdel-Gawad E, El-Ghannam D. Polymorphism of CYP1A1 gene and susceptibility to childhood acute lymphoblastic leukemia in Egypt. Leuk Lymphoma. 2014;55(3):618–23.
58. Zhuo W, Zhang L, Qiu Z, Zhu B, Chen Z. Does cytochrome P450 1A1 MspI polymorphism increase acute lymphoblastic leukemia risk? Evidence from 2013 cases and 2903 controls. Gene. 2012;510(1):14–21.
59. Park SK, Andreotti G, Sakoda LC, Gao YT, Rashid A, Chen J, et al. Variants in hormone-related genes and the risk of biliary tract cancers and stones: a population-based study in China. Carcinogenesis. 2009;30(4):606–14.
60. Hirata H, Hinoda Y, Okayama N, Suehiro Y, Kawamoto K, Kikuno N, et al. CYP1A1, SULT1A1, and SULT1E1 polymorphisms are risk factors for endometrial cancer susceptibility. Cancer. 2008;112(9):1964–73.
61. Esteller M, Garcia A, Martinez-Palones JM, Xercavins J, Reventos J. Germ line polymorphisms in cytochrome-P450 1A1 (C4887 CYP1A1) and methylenetetrahydrofolate reductase (MTHFR) genes and endometrial cancer susceptibility. Carcinogenesis. 1997;18(12):2307–11.
62. Zeng M, Lv Y, Wang HF, Yiguli HA, Zhang JR, Yisikandaer A. Correlation of CYP1A1 and GSTM1 gene polymorphisms and environmental factors to familial aggregation of esophageal cancer among the Kazakh ethnic group in Xinjiang. Genet Mol Res. 2015;14(4):19102–9.
63. Wang AH, Sun CS, Li LS, Huang JY, Chen QS. Relationship of tobacco smoking CYP1A1 GSTM1 gene polymorphism and esophageal cancer in Xi'an. World J Gastroenterol. 2002;8(1):49–53.
64. Shen FF, Zhou FY, Xue QS, Pan Y, Zheng L, Zhang H, et al. Association between CYP1A1 polymorphisms and esophageal cancer: a meta-analysis. Mol Biol Rep. 2013;40(10):6035–42.
65. Xu JL, Xia R, Sun L, Min X, Sun ZH, Liu C, et al. Association of CYP1A1 MspI polymorphism with oral cancer risk in Asian populations: a meta-analysis. Genet Mol Res. 2016;15(2).
66. Liu H, Jia J, Mao X, Lin Z. Association of CYP1A1 and GSTM1 Polymorphisms With Oral Cancer Susceptibility: A Meta-Analysis. Medicine (Baltimore). 2015;94(27):e895.
67. Gattás GJ, de Carvalho MB, Siraque MS, Curioni OA, Kohler P, Eluf-Neto J, et al. Genetic polymorphisms of CYP1A1, CYP2E1, GSTM1, and GSTT1 associated with head and neck cancer. Head Neck. 2006;28(9):819–26.
68. Cantlay AM, Lamb D, Gillooly M, Norrman J, Morrison D, Smith CA, et al. Association between the CYP1A1 gene polymorphism and susceptibility to emphysema and lung cancer. Clin Mol Pathol. 1995;48(4):M210–4.
69. Ezzeldin N, El-Lebedy D, Darwish A, El-Bastawisy A, Hassan M, Abd El-Aziz S, et al. Genetic polymorphisms of human cytochrome P450 CYP1A1 in an Egyptian population and tobacco-induced lung cancer. Genes Environ. 2017;39:7.
70. Zhan P, Wang Q, Qian Q, Wei SZ, Yu LK. CYP1A1 MspI and exon7 gene polymorphisms and lung cancer risk: an updated meta-analysis and review. J Exp Clin Cancer Res. 2011;30(1):99.
71. Acevedo C, Opazo JL, Huidobro C, Cabezas J, Iturrieta J, Quiñones Sepúlveda L. Positive correlation between single or combined genotypes of CYP1A1 and GSTM1 in relation to prostate cancer in Chilean people. Prostate. 2003;57(2):111–7.
72. Li H, Xiao D, Hu L, He T. Association of CYP1A1 polymorphisms with prostate cancer risk: an updated meta-analysis. Mol Biol Rep. 2012;39(12):10273–84.
73. Chen J, Cheng M, Yi L, Jiang CB. Relationship between CYP1A1 genetic polymorphisms and renal cancer in China. Asian Pac J Cancer Prev. 2011;12(9):2163–6.

74. Li H, Chen XL, Li HQ. Polymorphism of CYPIA1 and GSTM1 genes associated with susceptibility of gastric cancer in Shandong Province of China. World J Gastroenterol. 2005;11(37):5757–62.
75. Elderdery AY, Idris HME, Alruwaili SNL, Tebien EM, Alsrhani A, Alenazy FO, et al. The Influence of Genetic Polymorphisms in Cytochrome P450 (CYP1A1 and 2D6) Gene on the Susceptibility to Philadelphia Negative Chronic Myeloid Leukemia in Sudanese Patients. Int J Mol Sci. 2024;25(24).
76. Zeng W, Li Y, Lu E, Ma M. CYP1A1 rs1048943 and rs4646903 polymorphisms associated with laryngeal cancer susceptibility among Asian populations: a meta-analysis. J Cell Mol Med. 2016;20(2):287–93.
77. Kleine JP, Camargo-Kosugi CM, Carvalho CV, Silva FC, Silva ID. Analysis of CYP1A1 and COMT polymorphisms in women with cervical cancer. Genet Mol Res. 2015;14(4):18965–73.
78. Wang Y, Kong CZ, Zhang Z, Yang CM, Li J. Relationships between CYP1A1 genetic polymorphisms and bladder cancer risk: a meta-analysis. DNA Cell Biol. 2014;33(3):171–81.
79. Xu L, Wei H. Association between CYP1A1 2454A > G polymorphism and colorectal cancer risk: A meta-analysis. J Cancer Res Ther. 2015;11(4):760–4.
80. Sergentanis TN, Economopoulos KP. Four polymorphisms in cytochrome P450 1A1 (CYP1A1) gene and breast cancer risk: a meta-analysis. Breast Cancer Res Treat. 2010;122(2):459–69.
81. Shin A, Kang D, Choi JY, Lee KM, Park SK, Noh DY, et al. Cytochrome P450 1A1 (CYP1A1) polymorphisms and breast cancer risk in Korean women. Exp Mol Med. 2007;39(3):361–6.
82. Lu J, Zhao Q, Zhai YJ, He HR, Yang LH, Gao F, et al. Genetic polymorphisms of CYP1A1 and risk of leukemia: a meta-analysis. Onco Targets Ther. 2015;8:2883–902.
83. Hidaka A, Sasazuki S, Matsuo K, Ito H, Charvat H, Sawada N, et al. CYP1A1, GSTM1 and GSTT1 genetic polymorphisms and gastric cancer risk among Japanese: A nested case-control study within a large-scale population-based prospective study. Int J Cancer. 2016;139(4):759–68.
84. Vibhuti A, Arif E, Mishra A, Deepak D, Singh B, Rahman I, et al. CYP1A1, CYP1A2 and CYBA gene polymorphisms associated with oxidative stress in COPD. Clin Chim Acta. 2010;411(7-8):474–80.
85. Iqbal MS, Sardar N, Peng K, Almutairi LA, Duan X, Tanvir F, et al. Association between CYP1A2 gene variants -163 C/A (rs762551) and -3860 G/A (rs2069514) and bladder cancer susceptibility. BMC Cancer. 2024;24(1):880.
86. Watanabe J, Shimada T, Gillam EM, Ikuta T, Suemasu K, Higashi Y, et al. Association of CYP1B1 genetic polymorphism with incidence to breast and lung cancer. Pharmacogenetics. 2000;10(1):25–33.
87. Okobia MN, Bunker CH, Garte SJ, Zmuda JM, Ezeome ER, Anyanwu SN, et al. Cytochrome P450 1B1 Val432Leu polymorphism and breast cancer risk in Nigerian women: a case control study. Infect Agent Cancer. 2009;4 Suppl 1(Suppl 1):S12.
88. Chang BL, Zheng SL, Isaacs SD, Turner A, Hawkins GA, Wiley KE, et al. Polymorphisms in the CYP1B1 gene are associated with increased risk of prostate cancer. Br J Cancer. 2003;89(8):1524–9.
89. Zhang H, Li L, Xu Y. CYP1B1 polymorphisms and susceptibility to prostate cancer: a meta-analysis. PLoS One. 2013;8(7):e68634.
90. Jiang W, Sun G, Xiong J, Xi X, Shi Z. Association of CYP1B1 L432V polymorphism with urinary cancer susceptibility: a meta-analysis. Diagn Pathol. 2014;9:113.
91. Yu PJ, Chen WG, Feng QL, Chen W, Jiang MJ, Li ZQ. Association between CYP1B1 gene polymorphisms and risk factors and susceptibility to laryngeal cancer. Med Sci Monit. 2015;21:239–45.
92. Cauffiez C, Lo-Guidice JM, Quaranta S, Allorge D, Chevalier D, Cenée S, et al. Genetic polymorphism of the human cytochrome CYP2A13 in a French population: implication in lung cancer susceptibility. Biochem Biophys Res Commun. 2004;317(2):662–9.
93. Berköz M, Yalin S. Association of CYP2B6 G15631T polymorphism with acute leukemia susceptibility. Leuk Res. 2009;33(7):919–23.
94. Liu H, Li G, Sturgis EM, Shete S, Dahlstrom KR, Du M, et al. Genetic variants in CYP2B6 and HSD17B12 associated with risk of squamous cell carcinoma of the head and neck. Int J Cancer. 2022;151(4):553–64.

95. Yu LL, Zhang W, Li J, Zhao L. Association between CYP2B6 polymorphism and acute leukemia in a Han population of Northwest China. Mol Genet Genomic Med. 2020;8(5):e1162.
96. Qu W, Zhang F, Cheng Y, Li J, Zhou J. The impact of genetic variants in the CYP2C8 gene on bladder cancer susceptibility. Front Endocrinol (Lausanne). 2022;13:989030.
97. Yadav SS, Ruwali M, Pant MC, Shukla P, Singh RL, Parmar D. Interaction of drug metabolizing cytochrome P450 2D6 poor metabolizers with cytochrome P450 2C9 and 2C19 genotypes modify the susceptibility to head and neck cancer and treatment response. Mutat Res. 2010;684(1-2):49–55.
98. Uthansingh K, Parida PK, Pati GK, Sahu MK, Padhy RN. Evaluating the Association of Genetic Polymorphism of Cytochrome p450 (CYP2C9*3) in Gastric Cancer Using Polymerase Chain Reaction-Restriction Fragment Length Polymorphism (PCR-RFLP). Cureus. 2022;14(7):e27220.
99. Lim JS, Sutiman N, Muerdter TE, Singh O, Cheung YB, Ng RC, et al. Association of CYP2C19*2 and associated haplotypes with lower norendoxifen concentrations in tamoxifen-treated Asian breast cancer patients. Br J Clin Pharmacol. 2016;81(6):1142–52.
100. Chau TK, Marakami S, Kawai B, Nasu K, Kubota T, Ohnishi A. Genotype analysis of the CYP2C19 gene in HCV-seropositive patients with cirrhosis and hepatocellular carcinoma. Life Sci. 2000;67(14):1719–24.
101. Roddam PL, Rollinson S, Kane E, Roman E, Moorman A, Cartwright R, et al. Poor metabolizers at the cytochrome P450 2D6 and 2C19 loci are at increased risk of developing adult acute leukaemia. Pharmacogenetics. 2000;10(7):605–15.
102. Nida S, Javid B, Akbar M, Idrees S, Adil W, Ahmad GB. Gene variants of CYP1A1 and CYP2D6 and the risk of childhood acute lymphoblastic leukaemia; outcome of a case control study from Kashmir, India. Mol Biol Res Commun. 2017;6(2):77–84.
103. Wang N, Han JL, Mi YC, Xiao ZJ, Feng SZ, Zhou YL, et al. [Relationships between the gene polymorphisms of drug metabolizing enzymes and the outcome of the first induction chemotherapy in patients with de novo acute myeloid leukemia]. Zhongguo Shi Yan Xue Ye Xue Za Zhi. 2011;19(2):327–31.
104. Sobti RC, Onsory K, Al-Badran AI, Kaur P, Watanabe M, Krishan A, et al. CYP17, SRD5A2, CYP1B1, and CYP2D6 gene polymorphisms with prostate cancer risk in North Indian population. DNA Cell Biol. 2006;25(5):287–94.
105. Charoenchokthavee W, Panomvana D, Sriuranpong V, Areepium N. Prevalence of CYP2D6*2, CYP2D6*4, CYP2D6*10, and CYP3A5*3 in Thai breast cancer patients undergoing tamoxifen treatment. Breast Cancer (Dove Med Press). 2016;8:149–55.
106. Kim NH, Seol JE, Kim J, Lee BH, Hwang DY, Jeong J, et al. Red meat intake, CYP2E1 and PPARγ polymorphisms, and colorectal cancer risk. Eur J Cancer Prev. 2019;28(4):304–10.
107. Ye X, Peng T, Liu T, Su Z, Xiao K, Shang L, et al. [Association between aldehyde dehydrogenase-2/cytochrome P450 2E1 genetic polymorphism and habit of alcohol drinking and the susceptibility of hepatocellular carcinoma]. Wei Sheng Yan Jiu. 2010;39(1):42–5.
108. Ye X, Wang X, Shang L, Zhu G, Su H, Han C, et al. Genetic variants of ALDH2-rs671 and CYP2E1-rs2031920 contributed to risk of hepatocellular carcinoma susceptibility in a Chinese population. Cancer Manag Res. 2018;10:1037–50.
109. Fang Z, Wu Y, Zhang N. Association between CYP2E1 genetic polymorphisms and urinary cancer risk: a meta-analysis. Oncotarget. 2017;8(49):86853–64.
110. Ben Chaaben A, Abaza H, Douik H, Chaouch L, Ayari F, Ouni N, et al. [Genetic polymorphism of cytochrome P450 2E1 and the risk of nasopharyngeal carcinoma]. Bull Cancer. 2015;102(12):967–72.
111. Fernandes GM, Russo A, Proença MA, Gazola NF, Rodrigues GH, Biselli-Chicote PM, et al. CYP1A1, CYP2E1 and EPHX1 polymorphisms in sporadic colorectal neoplasms. World J Gastroenterol. 2016;22(45):9974–83.
112. Sameer AS, Nissar S, Qadri Q, Alam S, Baba SM, Siddiqi MA. Role of CYP2E1 genotypes in susceptibility to colorectal cancer in the Kashmiri population. Hum Genomics. 2011;5(6):530–7.

113. Datkhile KD, More AL, Patil MN, Gudur RA, Gudur AK. CYP2D6, CYP2E1 Gene Polymorphisms and Gastrointestinal Cancer Risk in Rural Maharashtra: A Hospital Based Case-Control Study. Asian Pac J Cancer Prev. 2024;25(9):3059–65.
114. Leng WD, Zeng XT, Chen YJ, Duan XL, Niu YM, Long RP, et al. Cytochrome P450 2E1 RsaI/PstI polymorphism and risk of esophageal cancer: A meta-analysis of 17 case-control studies. Exp Ther Med. 2012;4(5):938–48.
115. De Roos AJ, Rothman N, Inskip PD, Linet MS, Shapiro WR, Selker RG, et al. Genetic polymorphisms in GSTM1, -P1, -T1, and CYP2E1 and the risk of adult brain tumors. Cancer Epidemiol Biomarkers Prev. 2003;12(1):14–22.
116. Gervasini G, de Murillo SG, Ladero JM, Agúndez JA. CYP2W1 variant alleles in Caucasians and association of the CYP2W1 G541A (Ala181Thr) polymorphism with increased colorectal cancer risk. Pharmacogenomics. 2010;11(7):919–25.
117. Elsalahaty MI, Alkafaas SS, Bashir AO, El-Tarabily KA, El-Saadony MT, Yousef EH. Revealing the association between vitamin D metabolic pathway gene variants and lung cancer risk: a systematic review and meta-analysis. Front Genet. 2024;15:1302527.
118. He XF, Liu ZZ, Xie JJ, Wang W, Du YP, Chen Y, et al. Association between the CYP3A4 and CYP3A5 polymorphisms and cancer risk: a meta-analysis and meta-regression. Tumour Biol. 2014;35(10):9859–77.
119. Ali GT, Al-Azhary NM, Mokhtar DA. Frequency and prognostic significant of CYP3A4-A-290G polymorphism in acute myeloid leukemia. J Adv Res. 2014;5(6):657–61.
120. Sailaja K, Rao DN, Rao DR, Vishnupriya S. Analysis of CYP3A5*3 and CYP3A5*6 gene polymorphisms in Indian chronic myeloid leukemia patients. Asian Pac J Cancer Prev. 2010;11(3):781–4.
121. Liang Y, Han W, Yan H, Mao Q. Association of CYP3A5*3 polymorphisms and prostate cancer risk: A meta-analysis. J Cancer Res Ther. 2018;14(Supplement):S463–s7.
122. Rao DN, Manjula G, Sailaja K, Surekha D, Raghunadharao D, Rajappa S, et al. Association of CYP3A5*3 polymorphism with development of acute leukemia. Indian J Hum Genet. 2011;17(3):175–8.
123. Stone A, Ratnasinghe LD, Emerson GL, Modali R, Lehman T, Runnells G, et al. CYP3A43 Pro(340)Ala polymorphism and prostate cancer risk in African Americans and Caucasians. Cancer Epidemiol Biomarkers Prev. 2005;14(5):1257–61.
124. Yin Y, Tong L, Wan Z, Sui Y, Li F, Huang Q, et al. CYP4B1 polymorphisms and the risk of breast cancer in Chinese women: a case-control study. BMC Cancer. 2023;23(1):1177.
125. Yu S, Chen Z, Cheng J, Shi X, Liu J, Zhong P, et al. Case-control study on CYP4B1 gene polymorphism and susceptibility to gastric cancer in the chinese Han population. BMC Med Genomics. 2022;15(1):223.
126. Yang Y, Yuan S, Yan S, Dong K, Yang Y. Missense variants in CYP4B1 associated with increased risk of lung cancer among Chinese Han population. World J Surg Oncol. 2023;21(1):352.
127. Shi H, Zhang Y, Wang Y, Fang P, Liu Y. Genetic polymorphisms in CYP4F2 may be associated with lung cancer risk among females and no-smoking Chinese population. Front Oncol. 2023;13:1114218.
128. Li N, Shi H, Hou P, Gao L, Shi Y, Mi W, et al. Genetic variants of CYP4F12 gene are associated with glioma susceptibility. Int J Cancer. 2021;149(11):1910–5.
129. Wang B, Cao Z, Li Y, Zou H. Association between CYP17A1 rs743572 polymorphism and cancer risk: A meta-analysis. PLoS One. 2025;20(6):e0326843.
130. Skibola CF, Lightfoot T, Agana L, Smith A, Rollinson S, Kao A, et al. Polymorphisms in cytochrome P450 17A1 and risk of non-Hodgkin lymphoma. Br J Haematol. 2005;129(5):618–21.
131. Han JH, Lee YS, Kim HJ, Lee SY, Myung SC. Association between cytochrome CYP17A1, CYP3A4, and CYP3A43 polymorphisms and prostate cancer risk and aggressiveness in a Korean study population. Asian J Androl. 2015;17(2):285–91.
132. Hu MB, Xie W, Xiong B, Han DF, Li Y, Feng MH, et al. [Study on the relationship between polymorphisms of genes (CYP17, CYP19 and SULT1A1) and susceptibility to breast cancer in Chinese women]. Zhonghua Liu Xing Bing Xue Za Zhi. 2006;27(4):351–5.

133. Napoli N, Rastelli A, Ma C, Yarramaneni J, Vattikutti S, Moskowitz G, et al. Genetic polymorphism at Val80 (rs700518) of the CYP19A1 gene is associated with aromatase inhibitor associated bone loss in women with ER + breast cancer. Bone. 2013;55(2):309–14.
134. Gulyaeva LF, Mikhailova ON, PustyInyak VO, Kim IVt, Gerasimov AV, Krasilnikov SE, et al. Comparative analysis of SNP in estrogen-metabolizing enzymes for ovarian, endometrial, and breast cancers in Novosibirsk, Russia. Adv Exp Med Biol. 2008;617:359–66.
135. Wu IC, Zhao Y, Zhai R, Liu G, Ter-Minassian M, Asomaning K, et al. Association between polymorphisms in cancer-related genes and early onset of esophageal adenocarcinoma. Neoplasia. 2011;13(4):386–92.
136. Guo L, Liu Y, Liu L, Shao S, Cao Y, Guo J, et al. The CYP19A1 (TTTA)n Repeat Polymorphism May Affect the Prostate Cancer Risk: Evidence from a Meta-Analysis. Am J Mens Health. 2021;15(3):15579883211017033.
137. Zhang C, Cheng Y, Chen W, Li Q, Dai R, Wang Y, et al. Association of CYP19A1 rs28757157 polymorphism with lung cancer risk in the Chinese Han population. World J Surg Oncol. 2022;20(1):400.
138. Liang J, Li Y, Wan P, Zhang W, Han J, Zhang M, et al. CYP19A1 polymorphisms and bladder cancer risk in the Chinese Han population. Expert Rev Mol Diagn. 2024;24(8):743–52.
139. Al-Mukaynizi FB, Alanazi M, Al-Daihan S, Parine NR, Almadi M, Aljebreen A, et al. CYP19A1 gene polymorphism and colorectal cancer etiology in Saudi population: case-control study. Onco Targets Ther. 2017;10:4559–67.
140. Chang J, Zhong R, Tian J, Li J, Zhai K, Ke J, et al. Exome-wide analyses identify low-frequency variant in CYP26B1 and additional coding variants associated with esophageal squamous cell carcinoma. Nat Genet. 2018;50(3):338–43.
141. Blanke KL, Sacco JC, Millikan RC, Olshan AF, Luo J, Trepanier LA. Polymorphisms in the carcinogen detoxification genes CYB5A and CYB5R3 and breast cancer risk in African American women. Cancer Causes Control. 2014;25(11):1513–21.
142. Kikuchi A, Yamaya M, Suzuki S, Yasuda H, Kubo H, Nakayama K, et al. Association of susceptibility to the development of lung adenocarcinoma with the heme oxygenase-1 gene promoter polymorphism. Hum Genet. 2005;116(5):354–60.
143. Polonikov AV, Bushueva OY, Bulgakova IV, Freidin MB, Churnosov MI, Solodilova MA, et al. A comprehensive contribution of genes for aryl hydrocarbon receptor signaling pathway to hypertension susceptibility. Pharmacogenet Genomics. 2017;27(2):57–69.
144. Eskandari M, Awsat Mellati A, Mahmoodi K, Kamali K, Soltanpour MS. Association of the CYP1A1 rs4646903 polymorphism with susceptibility and severity of coronary artery disease. Mol Biol Res Commun. 2021;10(2):22–61.
145. Joy MS, Dornbrook-Lavender K, Blaisdell J, Hilliard T, Boyette T, Hu Y, et al. CYP2C9 genotype and pharmacodynamic responses to losartan in patients with primary and secondary kidney diseases. Eur J Clin Pharmacol. 2009;65(9):947–53.
146. Wu SN, Zhang Y, Gardner CO, Chen Q, Li Y, Wang GL, et al. Evidence for association of polymorphisms in CYP2J2 and susceptibility to essential hypertension. Ann Hum Genet. 2007;71(Pt 4):519–25.
147. Chen J, Wang DF, Fu GD, Ding J, Chen LY, Lv JL, et al. Meta-analysis of the association of the CYP2J2 G-50T polymorphism with coronary artery disease. Oncotarget. 2017;8(35):59618–27.
148. Wang SY, Xing PF, Zhang CY, Deng BQ. Association of CYP2J2 gene polymorphisms with ischemic stroke and stroke subtypes in Chinese population. Medicine (Baltimore). 2017;96(10):e6266.
149. Liu PY, Li YH, Chao TH, Wu HL, Lin LJ, Tsai LM, et al. Synergistic effect of cytochrome P450 epoxygenase CYP2J2*7 polymorphism with smoking on the onset of premature myocardial infarction. Atherosclerosis. 2007;195(1):199–206.
150. Vimaleswaran KS, Cavadino A, Berry DJ, Jorde R, Dieffenbach AK, Lu C, et al. Association of vitamin D status with arterial blood pressure and hypertension risk: a mendelian randomisation study. Lancet Diabetes Endocrinol. 2014;2(9):719–29.

151. Xu Z, Zhang D, Lin J, Li X, Liu Y, Gao J, et al. The influence of CYP2R1 polymorphisms and gene-obesity interaction with hypertension risk in a Chinese rural population. Nutr Metab Cardiovasc Dis. 2022;32(1):241–8.
152. Wang Q, Lin Z, Chen H, Ma T, Pan B. Effect of Cytochrome P450 Family 2 Subfamily R Member 1 Variants on the Predisposition of Coronary Heart Disease in the Chinese Han Population. Front Cardiovasc Med. 2021;8:652729.
153. Sedky NK, Abdel Rahman MF, Hassanein SI, Gad MZ. Genetic Variants of CYP2R1 Are Key Regulators of Serum Vitamin D Levels and Incidence of Myocardial Infarction in Middle-Aged Egyptians. Curr Pharm Biotechnol. 2018;19(3):265–73.
154. Xie Y, Acosta JN, Ye Y, Demarais ZS, Conlon CJ, Chen M, et al. Whole-Exome Sequencing Analyses Support a Role of Vitamin D Metabolism in Ischemic Stroke. Stroke. 2023;54(3):800–9.
155. Wang J, Ji H, Jia H, Guan D. Association between CYP3A4 gene rs4646437 polymorphism and the risk of hypertension in Chinese population: a case-control study. Biosci Rep. 2019;39(4).
156. He BX, Shi L, Qiu J, Tao L, Li R, Yang L, et al. A functional polymorphism in the CYP3A4 gene is associated with increased risk of coronary heart disease in the Chinese Han population. Basic Clin Pharmacol Toxicol. 2011;108(3):208–13.
157. Thompson EE, Kuttab-Boulos H, Witonsky D, Yang L, Roe BA, Di Rienzo A. CYP3A variation and the evolution of salt-sensitivity variants. Am J Hum Genet. 2004;75(6):1059–69.
158. Qi L, Liang W, Qiao H, Wang R, Han J, Xing X, et al. [Association of CYP2C19 and CYP3A5 gene polymorphisms with myocardial infarction]. Zhonghua Yi Xue Yi Chuan Xue Za Zhi. 2021;38(1):87–91.
159. Mayer B, Lieb W, Götz A, König IR, Aherrahrou Z, Thiemig A, et al. Association of the T8590C polymorphism of CYP4A11 with hypertension in the MONICA Augsburg echocardiographic substudy. Hypertension. 2005;46(4):766–71.
160. Yu K, Zhang T, Li X. Genetic role of CYP4A11 polymorphisms in the risk of developing cardiovascular and cerebrovascular diseases. Ann Hum Genet. 2018;82(6):370–81.
161. Zhang H, Jin L, Mu T, Fan Y, Zhang H, Zhu Y, et al. Associations of CYP4A11 gene-gene and gene-smoking interactions with essential hypertension in the male eastern Chinese Han population. Clin Exp Hypertens. 2017;39(5):448–53.
162. Sirotina S, Ponomarenko I, Kharchenko A, Bykanova M, Bocharova A, Vagaytseva K, et al. A Novel Polymorphism in the Promoter of the CYP4A11 Gene Is Associated with Susceptibility to Coronary Artery Disease. Dis Markers. 2018;2018:5812802.
163. Fu Z, Ma Y, Xie X, Huang D, Yang H, Nakayama T, et al. A novel polymorphism of the CYP4A11 gene is associated with coronary artery disease. Clin Appl Thromb Hemost. 2013;19(1):60–5.
164. Fu Z, Nakayama T, Sato N, Izumi Y, Kasamaki Y, Shindo A, et al. A haplotype of the CYP4A11 gene associated with essential hypertension in Japanese men. J Hypertens. 2008;26(3):453–61.
165. Shin DJ, Kwon J, Park AR, Bae Y, Shin ES, Park S, et al. Association of CYP2C19*2 and *3 genetic variants with essential hypertension in Koreans. Yonsei Med J. 2012;53(6):1113–9.
166. Bai Y, Huang R, Wan L, Zhao R. Association between CYP2C19 gene polymorphisms and lipid metabolism in Chinese patients with ischemic stroke. J Int Med Res. 2020;48(7):300060520934657.
167. Huang K, Ma T, Li Q, Zhong Z, Zhou Y, Zhang W, et al. Novel polymorphisms in CYP4A22 associated with susceptibility to coronary heart disease. BMC Med Genomics. 2024;17(1):66.
168. Wu Y, Zhao J, Zhao Y, Huang T, Ma X, Pang H, et al. Genetic variants in CYP4F2 were significantly correlated with susceptibility to ischemic stroke. BMC Med Genet. 2019;20(1):155.
169. Meng C, Wang J, Ge WN, Tang SC, Xu GM. Correlation between CYP4F2 gene rs2108622 polymorphism and susceptibility to ischemic stroke. Int J Clin Exp Med. 2015;8(9):16122–6.
170. Yu C, Yan Q, Fu C, Shi W, Wang H, Zeng C, et al. CYP4F2 genetic polymorphisms are associated with coronary heart disease in a Chinese population. Lipids Health Dis. 2014;13:83.
171. Huang D, Fu ZY, Yang YN, Xie X, Wang YH, Ma YT. [Association on the haplotypes of CYP4F2 gene and myocardial infarction]. Zhonghua Liu Xing Bing Xue Za Zhi. 2009;30(7):733–6.

172. Long F, Wang D, Su Q, Zhang Y, Li J, Xia S, et al. CYP4 subfamily V member 2 (CYP4V2) polymorphisms were associated with ischemic stroke in Chinese Han population. BMC Med Genomics. 2022;15(1):246.
173. Huang K, Ma T, Li Q, Zhong Z, Zhou Y, Zhang W, et al. CYP4V2 rs56413992 C > T was associated with the risk of coronary heart disease in the Chinese Han population: a case-control study. BMC Med Genomics. 2023;16(1):322.
174. Fu L, Zhao Y, Wu X, Liu H, Shi J, Lu J, et al. CYP7A1 genotypes and haplotypes associated with hypertension in an obese Han Chinese population. Hypertens Res. 2011;34(6):722–7.
175. Iwanicki T, Balcerzyk A, Niemiec P, Nowak T, Ochalska-Tyka A, Krauze J, et al. CYP7A1 gene polymorphism located in the 5' upstream region modifies the risk of coronary artery disease. Dis Markers. 2015;2015:185969.
176. Vargas-Alarcón G, Pérez-Méndez Ó, Posadas-Sánchez R, González-Pacheco H, Luna-Luna M, Escobedo G, et al. Associations of the CYP7A1 Gene Polymorphisms Located in the Promoter and Enhancer Regions with the Risk of Acute Coronary Syndrome, Plasma Cholesterol, and the Incidence of Diabetes. Biomedicines. 2024;12(3).
177. Dai CF, Xie X, Ma YT, Yang YN, Li XM, Fu ZY, et al. The relationship between the polymorphisms of the CYP17A1 gene and hypertension: A meta-analysis. J Renin Angiotensin Aldosterone Syst. 2015;16(4):1314–20.
178. Hou B, Jia X, Deng Z, Liu X, Liu H, Yu H, et al. Exploration of CYP21A2 and CYP17A1 polymorphisms and preeclampsia risk among Chinese Han population: a large-scale case-control study based on 5021 subjects. Hum Genomics. 2020;14(1):33.
179. Dai CF, Xie X, Yang YN, Li XM, Zheng YY, Fu ZY, et al. Relationship between CYP17A1 genetic polymorphism and coronary artery disease in a Chinese Han population. Lipids Health Dis. 2015;14:16.
180. Lu S, Zhong J, Zhang Y, Huang K, Wu M, Zhou Y, et al. CYP17A1 Polymorphisms Are Linked to the Risk of Coronary Heart Disease in a Case-Control Study. J Cardiovasc Pharmacol. 2019;74(2):98–104.
181. Bampali K, Grassos C, Mouzarou A, Liakos C, Mertzanos G, Lamnissou K, et al. Genetic Variant in the CYP19A1 Gene Associated with Coronary Artery Disease. Genet Res Int. 2015;2015:820323.
182. Meng Y, Adi D, Wu Y, Wang Y, Abudoukelimu M, Huang D, et al. CYP19A1 polymorphisms associated with coronary artery disease and circulating sex hormone levels in a Chinese population. Oncotarget. 2017;8(57):97101–13.
183. Dos Santos BR, Casanova G, da Silva TR, Oppermann K, Spritzer PM. Association between the aromatase (CYP19A1) gene variant rs10046 and cardiovascular risk in postmenopausal women. Arch Endocrinol Metab. 2024;68(Spec Issue):e240087.
184. Liang T, Zhang X, Liang A, Wu H, Wang Q, He J, et al. The effect of CYP7B1 polymorphisms on the risk of coronary heart disease in Hainan Han population. BMC Med Genomics. 2021;14(1):220.
185. Schillinger M, Exner M, Mlekusch W, Domanovits H, Huber K, Mannhalter C, et al. Heme oxygenase-1 gene promoter polymorphism is associated with abdominal aortic aneurysm. Thromb Res. 2002;106(2):131–6.
186. Ono K, Mannami T, Iwai N. Association of a promoter variant of the haeme oxygenase-1 gene with hypertension in women. J Hypertens. 2003;21(8):1497–503.
187. Esinler I, Aktas D, Otegen U, Alikasifoglu M, Yarali H, Tuncbilek E. CYP1A1 gene polymorphism and polycystic ovary syndrome. Reprod Biomed Online. 2008;16(3):356–60.
188. Elfaki I, Almutairi FM, Mir R, Khan R, Abu-duhier F. CYTOCHROME P450 CYP1B1*2 GENE AND ITS ASSOCIATION WITH T2D IN TABUK POPULATION, NORTHWESTERN REGION OF SAUDI ARABIA. Asian Journal of Pharmaceutical and Clinical Research. 2018;11(1):55–9.
189. Pu Y, Liu Q, Hu K, Liu X, Bai H, Wu Y, et al. CYP2E1 C-1054T and 96-bp I/D genetic variations and risk of gestational diabetes mellitus in chinese women: a case-control study. BMC Pregnancy Childbirth. 2023;23(1):403.
190. Mahdi F, Raza ST, Rizvi S, Abbas S, Karoli R. Distribution of Genetic Polymorphisms in Drug Metabolizing Gene Cytochrome P450 (CYP2C8*3 and CYP2C9*2) in a North Indian Type 2 Diabetes Population. Exploratory Research and Hypothesis in Medicine. 2016;1(3):42–6.

191. Ramos-Lopez E, Brück P, Jansen T, Herwig J, Badenhoop K. CYP2R1 (vitamin D 25-hydroxylase) gene is associated with susceptibility to type 1 diabetes and vitamin D levels in Germans. Diabetes Metab Res Rev. 2007;23(8):631–6.
192. Araújo E, Lima S, Galdino OA, Arrais RF, de Souza KSC, de Rezende AA. Association of CYP2R1 and VDR Polymorphisms with Metabolic Syndrome Components in Non-Diabetic Brazilian Adolescents. Nutrients. 2022;14(21).
193. Chang K, Chen Y, Xiong J, Ren J, Jiang Z. Association of cytochrome P450 2C19 *2 and *3 variants with type 2 diabetes mellitus in Chinese population. Journal of Xiangya Medicine. 2019;4.
194. Yamada Y, Matsuo H, Watanabe S, Kato K, Yajima K, Hibino T, et al. Association of a polymorphism of CYP3A4 with type 2 diabetes mellitus. Int J Mol Med. 2007;20(5):703–7.
195. Zhong S, Chèvre R, Castaño Mayan D, Corlianò M, Cochran BJ, Sem KP, et al. Haploinsufficiency of CYP8B1 associates with increased insulin sensitivity in humans. J Clin Invest. 2022;132(21).
196. Rezgoun ML, El Khour D, Bendaoud H, Chellat D. CYP17A1 (rs74357) polymorphism and polycystic ovary syndrome risk: a systemic review and meta-analysis. Acta Biomed. 2023;94(4):e2023167.
197. Xu X, Hu K, Shi H, Yu Y, Xu J, Sun Y. The single-nucleotide polymorphism rs743572 of CYP17A1 shows significant association with polycystic ovary syndrome: a meta-analysis. Reprod Biomed Online. 2021;43(5):941–51.
198. Wang L, Niu YM, Wu SS, Zhang C, Zhou L, Zuo HX, et al. A Study on the Association Between Polymorphisms in the Cytochrome P450 Family 17 Subfamily A Member 1 Gene Region and Type 2 Diabetes Mellitus in Han Chinese. Front Endocrinol (Lausanne). 2018;9:323.
199. Wang H, Li Q, Wang T, Yang G, Wang Y, Zhang X, et al. A common polymorphism in the human aromatase gene alters the risk for polycystic ovary syndrome and modifies aromatase activity in vitro. Mol Hum Reprod. 2011;17(6):386–91.
200. Long JR, Shu XO, Cai Q, Wen W, Kataoka N, Gao YT, et al. CYP19A1 genetic polymorphisms may be associated with obesity-related phenotypes in Chinese women. Int J Obes (Lond). 2007;31(3):418–23.
201. Martínez-Hernández A, Córdova EJ, Rosillo-Salazar O, García-Ortíz H, Contreras-Cubas C, Islas-Andrade S, et al. Association of HMOX1 and NQO1 Polymorphisms with Metabolic Syndrome Components. PLoS One. 2015;10(5):e0123313.
202. Wang CD, Chen N, Huang L, Wang JR, Chen ZY, Jiang YM, et al. Impact of CYP1A1 Polymorphisms on Susceptibility to Chronic Obstructive Pulmonary Disease: A Meta-Analysis. Biomed Res Int. 2015;2015:942958.
203. Cheng SL, Yu CJ, Yang PC. Genetic polymorphisms of cytochrome p450 and matrix metalloproteinase in chronic obstructive pulmonary disease. Biochem Genet. 2009;47(7-8):591–601.
204. Arif E, Vibhuti A, Alam P, Deepak D, Singh B, Athar M, et al. Association of CYP2E1 and NAT2 gene polymorphisms with chronic obstructive pulmonary disease. Clin Chim Acta. 2007;382(1-2):37–42.
205. Ding Y, Li Q, Feng Q, Xu D, Wu C, Zhao J, et al. CYP2B6 genetic polymorphisms influence chronic obstructive pulmonary disease susceptibility in the Hainan population. Int J Chron Obstruct Pulmon Dis. 2019;14:2103–15.
206. Polonikov AV, Ivanov VP, Solodilova MA. Genetic variation of genes for xenobiotic-metabolizing enzymes and risk of bronchial asthma: the importance of gene-gene and gene-environment interactions for disease susceptibility. J Hum Genet. 2009;54(8):440–9.
207. Shi H, Xu J, Feng Q, Sun J, Yang Y, Zhao J, et al. The effect of CYP3A4 genetic variants on the susceptibility to chronic obstructive pulmonary disease in the Hainan Han population. Genomics. 2020;112(6):4399–405.
208. Ding Y, Yang Y, Li Q, Feng Q, Xu D, Wu C, et al. The correlation between CYP4F2 variants and chronic obstructive pulmonary disease risk in Hainan Han population. Respir Res. 2020;21(1):86.
209. Siedlinski M, van Diemen CC, Postma DS, Boezen HM. Heme oxygenase 1 variations and lung function decline in smokers: proof of replication. J Med Genet. 2008;45(6):400.

210. Yamada N, Yamaya M, Okinaga S, Nakayama K, Sekizawa K, Shibahara S, et al. Microsatellite polymorphism in the heme oxygenase-1 gene promoter is associated with susceptibility to emphysema. Am J Hum Genet. 2000;66(1):187–95.
211. Guénégou A, Leynaert B, Bénessiano J, Pin I, Demoly P, Neukirch F, et al. Association of lung function decline with the heme oxygenase-1 gene promoter microsatellite polymorphism in a general population sample. Results from the European Community Respiratory Health Survey (ECRHS), France. J Med Genet. 2006;43(8):e43.
212. Sheu CC, Zhai R, Wang Z, Gong MN, Tejera P, Chen F, et al. Heme oxygenase-1 microsatellite polymorphism and haplotypes are associated with the development of acute respiratory distress syndrome. Intensive Care Med. 2009;35(8):1343–51.
213. Li J, Chen Y, Mo S, Nai D. Potential Positive Association between Cytochrome P450 1A1 Gene Polymorphisms and Recurrent Pregnancy Loss: a Meta-Analysis. Ann Hum Genet. 2017;81(4):161–73.
214. Singh A, Koner BC, Ray PC, Prasad S, Jamatia E, Masroor M, et al. Effect of CYP1A1 gene polymorphism and psychological distress on seminal analysis parameters. Reprod Health. 2016;13(1):60.
215. Barbosa AM, de Souza SR, Frare AB, Costa ESRC, da Costa IR, Freitas ESKS, et al. Association of CYP1A1 (cytochrome P450) MspI polymorphism in women with endometriosis. Genet Mol Res. 2016;15(3).
216. Painter JN, Nyholt DR, Krause L, Zhao ZZ, Chapman B, Zhang C, et al. Common variants in the CYP2C19 gene are associated with susceptibility to endometriosis. Fertil Steril. 2014;102(2):496–502.e5.
217. Tuo Y, He JY, Yan WJ, Yang J. Association between CYP19A1, GSTM1, GSTT1, and GSTP1 genetic polymorphisms and the development of endometriosis in a Chinese population. Genet Mol Res. 2016;15(4).
218. Cong L, Fu Q, Gao T. CYP17A1 rs743572 polymorphism might contribute to endometriosis susceptibility: evidences from a case-control study. Medicine (Baltimore). 2018;97(28):e11415.
219. Milewski Ł, Ścieżyńska A, Ponińska J, Soszyńska M, Barcz E, Roszkowski PI, et al. Endometriosis Is Associated with Functional Polymorphism in the Promoter of Heme Oxygenase 1 (HMOX1) Gene. Cells. 2021;10(3).
220. Martinez-Hernandez A, Perez-Guerrero EE, Macias-Islas MA, Nava-Valdivia CA, Villagomez-Vega A, Contreras-Haro B, et al. Polymorphisms CYP2R1 rs10766197 and CYP27B1 rs10877012 in Multiple Sclerosis: A Case-Control Study. J Immunol Res. 2021;2021:7523997.
221. Anwarullah, Aslam M, Badshah M, Abbasi R, Sultan A, Khan K, et al. Further evidence for the association of CYP2D6*4 gene polymorphism with Parkinson's disease: a case control study. Genes Environ. 2017;39:18.
222. Zhuo Q, Zhong X, Wen J, Ding Y. Association of CYP4A22 gene polymorphisms with cerebral stroke risk in the Chinese Han population. J Gene Med. 2023;25(8):e3515.
223. Xie L, Yan H, Shi L, Kong Y, Huang M, Li J, et al. Association between CYP17A1 rs3824755 and rs743572 gene polymorphisms and Alzheimer's disease in the Chinese Han population. Neurosci Lett. 2016;618:77–82.
224. Zheng J, Yan H, Shi L, Kong Y, Zhao Y, Xie L, et al. The CYP19A1 rs3751592 variant confers susceptibility to Alzheimer disease in the Chinese Han population. Medicine (Baltimore). 2016;95(35):e4742.
225. Kölsch H, Lütjohann D, Jessen F, Popp J, Hentschel F, Kelemen P, et al. CYP46A1 variants influence Alzheimer's disease risk and brain cholesterol metabolism. Eur Psychiatry. 2009;24(3):183–90.
226. He XM, Zhang ZX, Zhang JW, Zhou YT, Wu CB, Tang MN, et al. An intronic CYP46A1 polymorphism is associated with Alzheimer disease in a Chinese Han population. J Mol Neurosci. 2012;47(3):514–8.
227. Jia F, Liu Z, Song N, Du X, Xie J, Jiang H. The association between CYP46A1 rs4900442 polymorphism and the risk of Alzheimer's disease: A meta-analysis. Neurosci Lett. 2016;620:83–7.
228. Yen JH, Tsai WC, Lin CH, Ou TT, Hu CJ, Liu HW. Cytochrome P450 1A1 and manganese superoxide dismutase genes polymorphisms in reactive arthritis. Immunol Lett. 2003;90(2-3):151–4.
229. Yen JH, Tsai WC, Lin CH, Ou TT, Hu CJ, Liu HW. Cytochrome p450 1Al gene polymorphisms in patients with psoriatic arthritis. Scand J Rheumatol. 2004;33(1):19–23.

230. Yilmaz MB, Pazarbasi A, Guzel AI, Kocaturk-Sel S, Kasap H, Kasap M, et al. Association of serum sex steroid levels and bone mineral density with CYP17 and CYP19 gene polymorphisms in postmenopausal women in Turkey. Genet Mol Res. 2011;10(3):1999–2008.
231. Xiong DH, Shen H, Zhao LJ, Xiao P, Yang TL, Guo Y, et al. Robust and comprehensive analysis of 20 osteoporosis candidate genes by very high-density single-nucleotide polymorphism screen among 405 white nuclear families identified significant association and gene-gene interaction. J Bone Miner Res. 2006;21(11):1678–95.
232. Napoli N, Rini GB, Serber D, Giri T, Yarramaneni J, Bucchieri S, et al. The Val432Leu polymorphism of the CYP1B1 gene is associated with differences in estrogen metabolism and bone density. Bone. 2009;44(3):442–8.

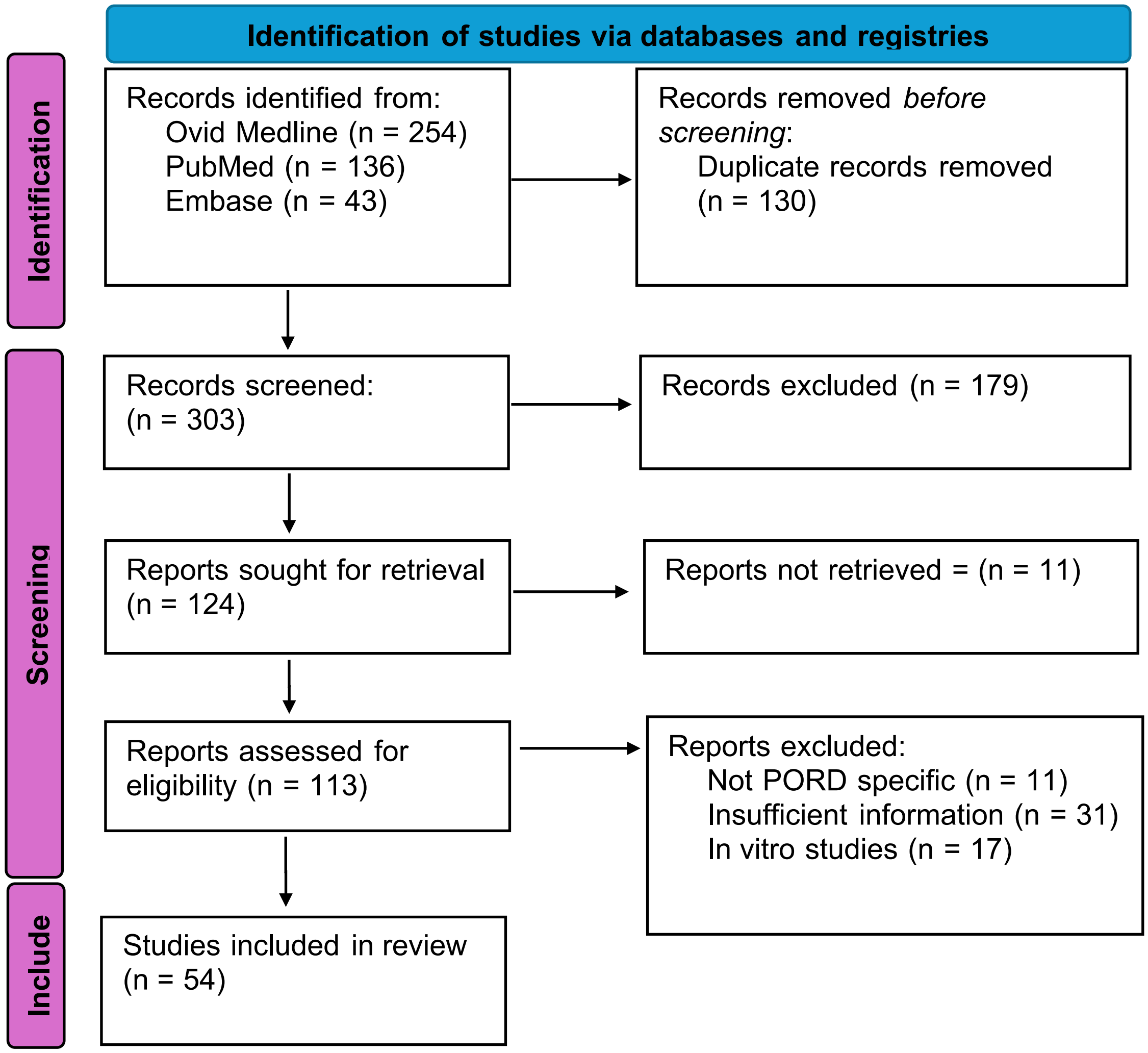

Suppl Figure S1. Selection process of the scoping review following the Preferred Reporting Items for Systematic Reviews and Meta-Analyses (PRISMA) guidelines (*DOI: 10.7326/M18-0850*). In brief: Articles were included if: 1) the patients had genetically confirmed biallelic P450 oxidoreductase deficiency, 2) the research discussed clinical outcomes, associated morbidities or long-term health outcomes linked to PORD, 3) they were original research articles, clinical or observational studies, case reports, narrative or systematic reviews, and 4) they were published between 2004 and September 2025 in English or with an English translation available. Articles were excluded if: 1) they focused primarily on other disorders of sex development without specific mention or focus on PORD, 2) if the articles were based solely on animal models or in vitro research without direct clinical relevance to human PORD, or 3) when clinically relevant data could not be reliably attributed to individual patients.

PRISMA Checklist of Scoping Review Manuscript

PRISMA-ScR Item	Checklist Requirement	Location in Revised Manuscript
**TITLE**	Identifies the report as a scoping review	Title page: "Long-term health outcomes and comorbidities in cytochrome P450 oxidoreductase (POR) deficiency: a scoping review of evidence and knowledge gaps"
**ABSTRACT**	Structured summary of background, objectives, methods, results	Abstract (full section)
**INTRODUCTION – Rationale**	Explains why a scoping review is appropriate	Introduction, final paragraph: "Given these uncertainties, a comprehensive mapping... A scoping review is particularly suited..."
**INTRODUCTION – Objectives**	States review objectives	Introduction, final paragraph: "This scoping review therefore aims to..."
**METHODS – Protocol & Registration**	States whether a protocol exists	Methods, first paragraph (no protocol registered)
**METHODS – Eligibility Criteria**	Clear inclusion/exclusion criteria	Methods, paragraphs 3–4: "Articles were included if..." and "Articles were excluded if..."
**METHODS – Information Sources**	Databases, dates, search limits	Methods, paragraphs 1–2: Ovid MEDLINE, Embase, PubMed; search date 16 Sept 2025
**METHODS – Search Strategy**	Full search strategy for at least one database	Methods, paragraphs 1–2: detailed search terms for MEDLINE, Embase, PubMed
**METHODS – Selection of Sources**	Screening process, number of reviewers	Methods, paragraph 5: "One author screened... assessed together with a second author..."
**METHODS – Data Charting Process**	How data were extracted	Methods, paragraph 6: "Data from all eligible publications were extracted and tabulated..."
**METHODS – Data Items**	Variables extracted	Methods, paragraph 6: age, karyotype, genotype, adrenal function, DSD, puberty, skeletal findings, comorbidities
**METHODS – Critical Appraisal (optional)**	Whether appraisal was done	Not performed
**METHODS – Synthesis of Results**	How results were summarized	Methods, paragraphs 6–8: phenotype matrix, heatmap, LitVar2/OMIM queries
**RESULTS – Selection of Sources**	Flow diagram, number of included studies	Results, first paragraph: "54 eligible articles... 136 patients..."; PRISMA flow chart in Suppl Figure S1
**RESULTS – Characteristics of Sources**	Summary of included studies	Results, section "Characteristic clinical profile..."

**RESULTS – Critical Appraisal Within Sources (optional)**	If done, present results	Not applicable
**RESULTS – Results of Individual Sources**	Key findings from each included source	Results sections on reproductive, skeletal, cardiovascular, neurocognitive, drug metabolism
**RESULTS – Synthesis of Results**	Overall patterns and themes	Results, entire section; Figures 1–3
**DISCUSSION – Summary of Evidence**	Interpretation of findings	Gaps and future directions, first paragraphs
**DISCUSSION – Limitations**	Limitations of evidence and review process	Gaps and future directions, final paragraphs: “Despite increasing recognition… evidence remains sparse…”
**DISCUSSION – Conclusions**	Implications and future directions	Gaps and future directions, final paragraph
**FUNDING**	Funding sources	Acknowledgements / Funding statement
